# The conserved outer mitochondrial membrane protein Mtch regulates mitophagy during *Drosophila* intestinal development

**DOI:** 10.1371/journal.pbio.3003616

**Published:** 2026-01-23

**Authors:** Lucas J. Restrepo, Jasmine K. Graslie, Tina M. Fortier, Eric H. Baehrecke

**Affiliations:** Department of Molecular, Cell and Cancer Biology, University of Massachusetts Chan Medical School, Worcester, Massachusetts, United States of America; Institute of Basic Medical Sciences, NORWAY

## Abstract

The clearance of mitochondria by autophagy (mitophagy) is important for cell health. Mutations in genes that are required for mitophagy, including *Vps13D*, *PINK1*, and *Parkin*, are associated with movement disorders, but gaps exist in our understanding of how Vps13D regulates mitophagy. Here, we identify Mtch (MTCH2 in humans) as a regulator of mitophagy based on a relationship with Vps13D during developmentally programmed mitophagy in *Drosophila* intestine enterocyte cells. Similar to *Vps13D* mutant cells, *Mtch* mutant cells fail to clear mitochondria and possess elevated markers of autophagy. Genetic and molecular experiments reveal that Mtch and Vps13D function in a mitophagy pathway with PINK1, Parkin, and the mitophagy receptor BNIP3. Unlike *Vps13D* and *Parkin* mutant cells, *Mtch* is required for proper expression of the tail-anchored protein BNIP3. Thus, the tail-anchored protein insertase function of Mtch/MTCH2 likely explains how these proteins possess multiple cell context-specific functions.

## Abstract 2

Mitophagy is critical for cellular health and is associated with multiple diseases. This study shows that the outer mitochondrial membrane protein Mtch functions as a novel regulator of mitophagy during Drosophila intestinal development, likely by regulating the insertion of the mitophagy receptor BNIP3.

## Introduction

Macroautophagy (self-eating, and hereafter autophagy) is an important cytoplasmic recycling process that is critical for cell and organism health [1]. During autophagy, cytoplasmic cargoes are enveloped in an autophagosome and trafficked to lysosomes for degradation [2]. Autophagy can either clear nonspecific cargoes or selectively clear specific cargoes, including the organelles mitochondria, endoplasmic reticulum (ER), and peroxisomes [3]. Alterations in mitochondrial-selective autophagy (mitophagy) have been associated with multiple diseases, including Parkinson’s, Alzheimer’s, and other diseases [4]. Two of the most frequently mutated genes in familial Parkinson’s disease are *PINK1* and *Parkin*, and these two genes are key regulators of mitophagy [5]. Interestingly, PINK1, Parkin, and the mitophagy receptor BNIP3 are each required for the removal of mitochondria in the *Drosophila* intestine [6,7], but other studies suggest that they function in distinct mitophagy pathways [8]. Thus, it is important to understand the relationship between PINK1, Parkin, and BNIP3 function in mitophagy, and determine how they integrate to clear mitochondria in the physiological setting of the *Drosophila* intestine during development.

Vps13D, an evolutionarily conserved lipid transfer and ubiquitin binding protein, is required for mitochondrial clearance during *Drosophila* intestine development and has been implicated in a pediatric movement disorder [9–11]. Structural studies of Vps13D have revealed that it functions as a lipid conduit between membranes, where a hydrophobic groove acts as a bridge for lipid supply [12]. Vps13D regulates mitochondrial and ER contacts, where it physically interacts with the ER scramblase Vmp1 [13]. Thus, investigating how Vps13D functions at the mitochondrial membrane is essential to understanding its function during mitophagy. Importantly, proteomics analysis revealed that Vps13D physically interacts with multiple outer mitochondrial membrane proteins [14].

MTCH2 is an outer mitochondrial membrane protein with multiple roles in cell health. Originally defined as a regulator of cell death, MTCH2 was characterized to be important in tBID-mediated apoptosis [15]. MTCH2 has also been studied in the context of lipid metabolism, where it is an important regulator of de novo lipogenesis and mitochondrial fusion [16]. Most recently, MTCH2 was described to function in tail-anchored protein insertion into the outer mitochondrial membrane as a mitochondrial protein insertase [17]. Despite extensive investigation, it remains unclear how MTCH2 can play multiple roles in mitochondrial and cell health.

MTCH2 physically interacts with VPS13D in human cells [14]. This prompted our investigation of the relationship between Vps13D and the MTCH2 orthologue Mtch in *Drosophila*. Here, we identify Mtch as a regulator of mitochondrial clearance during *Drosophila* intestine development. We show that Mtch and Vps13D function in the same genetic pathway to regulate mitophagy. Importantly, Mtch also functions in a pathway with PINK1, Parkin, and BNIP3. Our data support a model with Mtch functioning as a BNIP3 insertase that is required for mitophagy downstream of PINK1 and Parkin, and this insertase function can potentially explain the diverse cellular functions of MTCH family proteins.

## Results

### Mtch regulates mitochondrial clearance by autophagy during development

**Fig 1:**
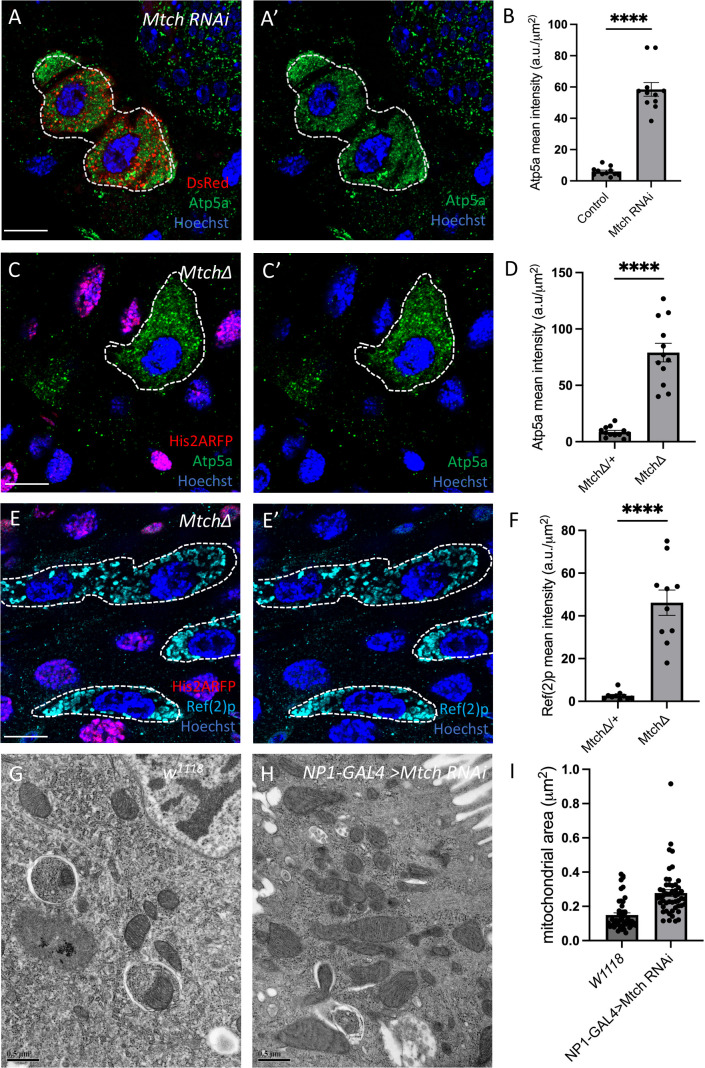
*Mtch* is required for mitochondrial clearance. (A and A’) *Mtch* RNAi (v106996) knockdown cells (DsRed labeled, white dotted outline) accumulate the mitochondrial protein Atp5a (green). Scale bar is 20 μm. **(B)** Quantification of Atp5a fluorescence intensity in *Mtch* knockdown and control cells (*n* = 11 *Mtch* and *n* = 11 control cells across 3 independent animals were measured). Data are presented as mean ± SEM. ****p < 0.0001. (C and C’) *Mtch* mutant cells (absence of nuclear RFP, white dotted outline) accumulate the mitochondrial protein Atp5a (green). Scale bar is 20 μm. **(D)** Quantification of Atp5a fluorescence intensity in mutant and control cells (*n* = 12 *Mtch* and *n* = 12 control cells across 3 independent animals were measured). Data are presented as mean ± SEM. ****p < 0.0001. (E and E’) *Mtch* mutant cells (absence of nuclear RFP, white dotted outline) accumulate autophagy adaptor Ref(2)p (cyan). Scale bar is 20 μm. **(F)** Quantification of Ref(2)p fluorescence intensity in *Mtch* mutant and control cells (*n* = 10 knockout and *n* = 10 control cells across 3 independent animals were measured). Data are presented as mean ± SEM. ***p < 0.001. **(G)** Electron micrograph of *w*^*1118*^ control enterocyte in the *Drosophila* intestine 2 h after puparium formation. Scale bar is 0.5 μm. **(H)** Electron micrograph of enterocyte cell-specific *Mtch* RNAi in the *Drosophila* intestine at 2 h after puparium formation. Scale bar is 0.5 μm. **(I)** Quantification of mitochondrial area of *Mtch* knockdown intestine cells compared to control. (*n* = 50 mitochondria across 3 separate experiments). Data are presented as mean ± SEM. ****p < 0.0001. The underlying data can be found in S1 Data.

Vps13D is required for the clearance of mitochondria in the developing *Drosophila* intestine, but the proteins on the mitochondrial surface that interact with and have similar function to *Vps13D* are poorly understood. Therefore, we screened a group of genes known to encode mitochondrial proteins that physically interact with VPS13D in human cells for phenotypes that are similar to *Vps13D* in enterocytes of the *Drosophila* intestine (S1 Table [14]). We examined markers of autophagy and mitochondrial clearance defects, including Ref(2)p, Atg8a, and Atp5a accumulation, in cells expressing RNAi to knockdown gene function 2 h after puparium formation (2 h APF) at a stage soon after the induction of mitophagy. The most robust defects in mitochondrial Atp5a clearance were observed in *Mtch* RNAi knockdown cells that are marked by the presence of red fluorescent protein (RFP) compared to neighboring control cells (Fig 1A, 1B). In addition, *Mtch* RNAi knockdown cells also exhibited accumulations of the autophagy adaptor protein Ref(2)p and core autophagy protein Atg8a compared to control neighboring cells (S1A–S1D Fig). Importantly, a second independent RNAi strain that targets *Mtch* resulted in the same accumulation of Atp5a and Ref(2)p in intestine enterocytes (S1E–S1F’ Fig). Thus, knockdown of *Mtch* phenocopies *Vps13D* mutant defects in enterocytes of the developing midgut. To confirm the *Mtch* RNAi phenotypes, we used CRISPR-Cas9 gene editing to delete the Mtch open reading frame and generate a null mutant allele. Similar to RNAi knockdown, *Mtch* null mutant enterocyte cells accumulate Atp5a compared to neighboring control cells 2 h APF (Fig 1C and 1D). *Mtch* mutant intestine cells also possess elevated cytoplasmic levels of the autophagy substrate Ref(2)p and the core autophagy protein Atg8a (Figs 1E–1H and S1G–S1H). Combined, these data indicate that *Mtch* mutant cells exhibit similar phenotypes to intestine enterocyte cells lacking *Vps13D* function.

We next investigated the function of *Mtch* in mitophagy by expressing the tandem GFP-RFP Mito-QC transgene in intestine enterocytes. The Mito-QC construct contains a portion of the mitochondrial tail-anchored protein Fis1 that enables association with mitochondria so that mitochondrial clearance can be followed during mitophagy. When autophagosomes fuse with acidic lysosomes pH-sensitive GFP signal is lost, and when mitophagy is dysfunctional GFP persists. Importantly, RFP signal is present in both autophagosomes and acidic autolysosomes. We tested Mito-QC in *Mtch* mutant cells and found that both GFP and RFP signal were decreased compared to control neighboring cells even when the signal in control cells was saturated (S2A–S2A” Fig). We also used a Mito-GFP construct that is associated with mitochondria because of mitochondrial COX8 sequence, and observed a loss of GFP in *Mtch* mutant cells compared to control neighboring cells (S2B–S2B’ Fig). These results prompted analysis of the influence of *Mtch* on mitochondria using Transmission Electron Microscopy (TEM) in intestine enterocytes 2 h APF. Homozygous mutant *Mtch* animals are lethal during development. Thus, we expressed *Mtch* RNAi in the enterocytes of the intestine. *Mtch* knockdown intestine cells exhibited increased numbers of mitochondria compared to control *w*^*1118*^ cells (Fig 1G–1I). Consistent with both TEM and Atp5a immuno-staining data, intestine enterocytes with reduced *Mtch* function possess both elevated COX4 antigen and more mitochondria that possess membrane potential based on TMRE staining (S2C–S2F Fig). In addition, *Mtch* mutant intestine cells also exhibited equivalent amounts of cleaved caspase-3 staining as neighboring control cells (S2G–S2G’ Fig), suggesting that caspases are not causing mitochondrial content differences. These data reveal that widely used mitophagy sensors may not always provide an accurate reflection of mitochondrial clearance, as Atp5a, COX4, TEM, and TMRE analyses revealed the presence of mitochondria in *Mtch* mutant cells. In addition, these data reveal that *Mtch* has a previously unknown role in mitochondrial clearance, and that *Mtch* loss-of-function phenotypes in the intestine enterocytes are similar to *Vps13D* mutant cells.

### Mtch regulates Vps13D-dependent mitophagy

We next investigated the relationship between Mtch and Vps13D during developmentally programmed mitophagy in *Drosophila* intestine enterocytes. Antibodies do not exist to detect Mtch in *Drosophila*. Therefore, CRISPR-Cas9 gene editing was used to make an in-frame insertion of a 2x-HA epitope with the Mtch open reading frame on the C-terminus of the protein (S3A Fig). Mtch-2xHA is expressed in enterocytes of the intestine, and co-localizes with mitochondrial Atp5a (S3B–S3B” Fig). We confirmed that knockdown of *Mtch* by expression of RNAi in DsRed-expressing enterocytes significantly reduced Mtch-2xHA levels compared to neighboring control cells (S3C, S3D Fig). Interestingly, high resolution analysis of Mtch and a previously characterized endogenously tagged Vps13D-3xFLAG [13] revealed limited co-localization of these proteins (S3E Fig). Importantly, we observed that Mtch localizes in rings that are likely the outer mitochondrial membranes, and that multiple points of contact exist with Vps13D puncta with substantial lack of co-localization (S3F and S3G Fig). These results suggest that the relationship between Mtch and Vps13D may be dynamic, and raise questions about their direct relationship in a mitophagy pathway.

**Fig 2:**
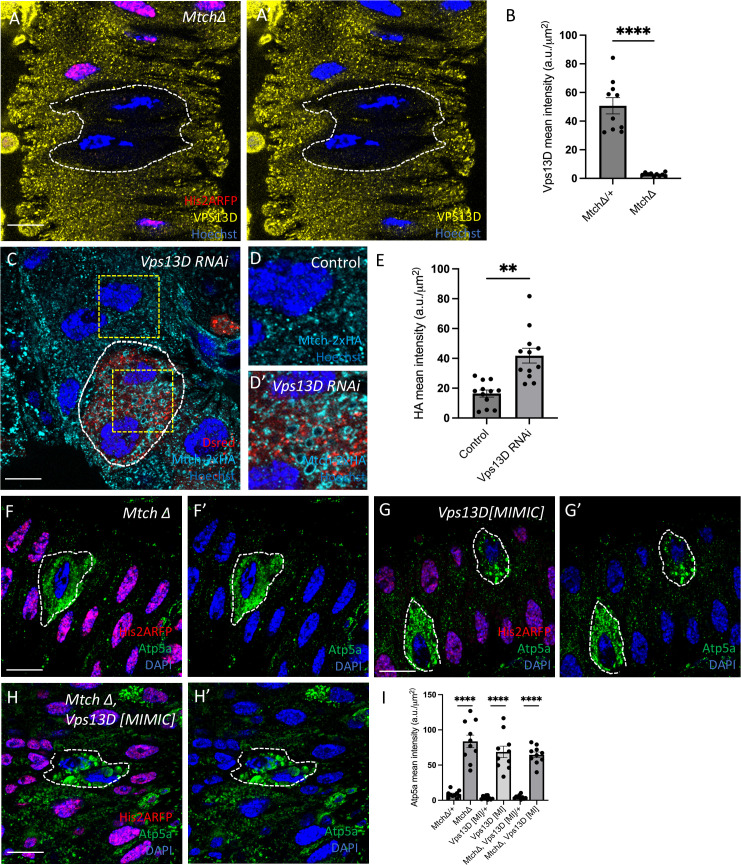
Relationship between Mtch and Vps13D during mitophagy. (A and A’) *Mtch* mutant cells (absence of nuclear RFP, white dotted outline) have decreased levels of Vps13D protein (yellow) in the developing *Drosophila* intestine. Scale bar is 20 μm. **(B)** Quantification of Vps13D fluorescence intensity in mutant and control cells (*n* = 10 *Mtch* mutant and *n* = 10 control cells across 3 independent animals were measured). Data are presented as mean ± SEM. ****p < 0.0001. **(C)** *Vps13D* RNAi knockdown cells (Dsred labeled, dotted line) have increased Mtch-2xHA levels (cyan). Scale bar is 20 μm. **(D)** Enlargement of Mtch-2xHA in control cell. Scale bar is 5 μm. (D’) Enlargement of Mtch-2xHA levels in *Vps13D* knockdown cell showing large rings of Mtch compared to control (D). **(E)** Quantification of Mtch-2xHA fluorescence intensity in *Vps13D* knockdown and control cells (*n* = 12 *Vps13D* knockdown and *n* = 12 control cells across 3 independent animals were measured). Data are presented as mean ± SEM. **p < 0.01. (F and F’) *Mtch* mutant cells (absence of nuclear RFP labeled, white dotted outline) inhibit clearance of mitochondrial protein Atp5a (green) compared to neighboring controls (nuclear RFP labeled). Scale bar is 20 μm. (G and G’) *Vps13D* mutant cells (absence of nuclear RFP labeled, dotted line) inhibit clearance of mitochondrial protein Atp5a (green) compared to neighboring controls (nuclear RFP labeled). Scale bar is 20 μm. (H and H’) *Mtch Vps13D* double mutant cells (absence of nuclear RFP labeled, dotted line) inhibit clearance of mitochondrial protein Atp5a (green) compared to neighboring control cells (nuclear RFP labeled). Scale bar is 20 μm. **(I)** Quantification of Atp5a mean fluorescence intensity in *Mtch, Vps13D*, and *Mtch Vps13D* knockout cells (*n* = 10 for each single and double mutant, and *n* = 10 control cells across 3 independent animals were measured). Data are presented as mean ± SEM. ****p < 0.0001. The underlying data can be found in S1 Data.

The similarity of Mtch and Vps13D phenotypes, despite limited co-localization (S3F and S3G Fig), prompted us to investigate if their loss influences the levels and localization of one another. We examined the influence of *Mtch* loss-of-function mutant cells that were stained with an antibody against Vps13D [9]. Vps13D protein puncta levels decreased dramatically in *Mtch* mutant cells compared to control neighboring intestine cells 2 h APF (Fig 2A, 2B). Importantly, *Vps13D* RNA levels are similar in intestine enterocytes of control and *Mtch* knockdown (S3H Fig), indicating that the change in Vps13D is at the level of the protein. Reciprocally, *Vps13D* function was reduced by expression of RNAi in cells that were marked with DsRed and stained for Mtch-2xHA. Mtch levels were slightly increased in *Vps13D* knockdown compared to neighboring control intestine cells (Fig 2C–2E). In addition, *Vps13D* knockdown cells exhibit large rings of Mtch compared to control neighboring cells (Fig 2D and 2D’). These data indicate that Mtch is required for proper Vps13D protein levels, and that *Vps13D* also influences Mtch abundance and distribution in enterocytes.

To further explore the genetic relationship between *Mtch* and *Vps13D*, we performed single and double mutant analysis to compare the levels of mitochondrial Atp5a and the autophagy protein Atg8a in enterocytes. Homozygous *Mtch* and *Vps13D* single mutant cells each exhibited elevated levels of Atp5a compared to neighboring control enterocytes 2 h APF (Fig 2F, 2G, and 2I). In addition, *Mtch Vps13D* double mutant cells did not exhibit an additive change in Atp5a levels compared to single mutants (Fig 2F–2I), indicating that *Mtch* and *Vps13D* function in the same mitochondrial clearance pathway. Analysis of Atg8a in *Mtch* and *Vps13D* single and double mutants did not reveal a significant difference in Atg8a levels (S4A–S4D Fig). Combined, these data indicate that *Vps13D* and *Mtch* function in the same mitophagy pathway.

### Mtch functions in a pathway with PINK1, Parkin, and BNIP3

**Fig 3:**
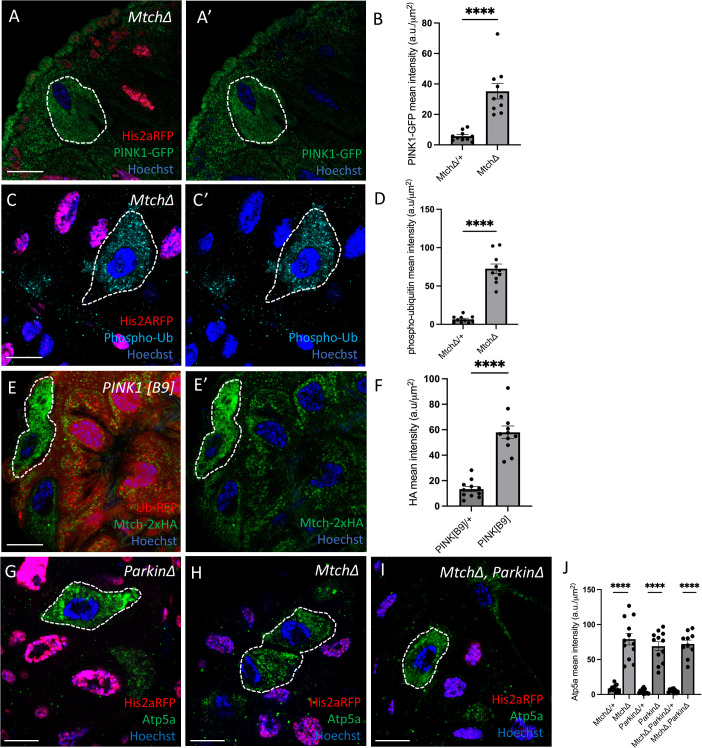
Mtch, PINK1, and Parkin function in a mitophagy pathway. (A and A’) *Mtch* mutant cells (absence of nuclear RFP labeled, dotted line) exhibit increased levels of PINK1-GFP (green) compared to control cells (nuclear RFP labeled). Scale bar is 20 μm. **(B)** Quantification of PINK-GFP mean fluorescence intensity in *Mtch* mutant vs. control cells (*n* = 10 *Mtch* mutant and *n* = 10 control cells across 3 independent animals were measured). Data are presented as mean ± SEM. ****p < 0.0001. (C and C’) *Mtch* mutant cells (absence of nuclear RFP labeled, dotted line) exhibit increased levels of phosphorylated ubiquitin (cyan) compared to control cells (nuclear RFP labeled). Scale bar is 20 μm. **(D)** Quantification of phosphorylated ubiquitin mean fluorescence intensity in mutant vs. control cells (*n* = 10 *Mtch* mutant and *n* = 10 control cells across 3 independent animals were measured). Data are presented as mean ± SEM. ****p < 0.0001. (E and E’) *PINK1* mutant cells (absence of RFP labeled, dotted line) exhibit increased levels of Mtch-2xHA (green) compared to control cells (RFP labeled). Scale bar is 20 μm. **(F)** Quantification of Mtch-2xHA mean fluorescence intensity in PINK1 in mutant vs. control cells (*n* = 10 mutant and *n* = 10 control cells across 3 independent animals were measured). Data are presented as mean ± SEM. ****p < 0.0001. **(G)** *Parkin* mutant cells (absence of nuclear RFP labeled, dotted line) inhibit clearance of mitochondrial protein Atp5a (green). Scale bar is 20 μm. **(H)** *Mtch* mutant cells (absence of nuclear RFP labeled, dotted line) inhibit clearance of mitochondrial protein Atp5a (green). Scale bar is 20 μm. **(I)** *Mtch* and *Parkin* double mutant cells (absence of nuclear RFP labeled, dotted line) inhibit clearance of mitochondrial protein Atp5a (green). Scale bar is 20 μm. **(J)** Quantification of Atp5a mean fluorescence intensity in mutant vs. control cells (*n* = 10–12 mutant and *n* = 10–12 control cells across 3 independent animals were measured). Data are presented as mean ± SEM. ****p < 0.0001. The underlying data can be found in S1 Data.

Our data indicates that Mtch and Vps13D function in the same mitophagy pathway. Thus, we next investigated the relationship between *Mtch* and previously known regulators of mitophagy in the *Drosophila* intestine during development, including PINK1, Parkin, and the mitophagy receptor BNIP3. Interestingly, PINK1 levels were significantly increased in *Mtch* mutant enterocytes compared to neighboring control cells 2 h APF (Fig 3A, 3B). *Pink1* is required for phosphorylation of ubiquitin and clearance of mitochondria in enterocytes [7]. Consistent with elevated PINK1 levels, we detected significantly increased levels of phosphorylated ubiquitin in *Mtch* mutant intestine cells compared to control enterocytes (Fig 3C, 3D). We also observed increased Mtch protein levels in *PINK1* mutant compared to control intestine cells 2 h APF (Fig 3E, 3F), likely because of the failure to clear mitochondria in *Pink1* mutant cells. These data suggest *Mtch* and *Pink1* function in the same mitophagy pathway.

Pink1 phosphorylates both ubiquitin and Parkin to regulate mitophagy [5], and Parkin is required for proper mitophagy in *Drosophila* intestine enterocytes [7]. Therefore, we investigated the relationship between Mtch and Parkin during mitophagy in the developing intestine. Importantly, single and double mutant analysis revealed similar elevated levels of mitochondrial Atp5a in *Mtch* and *Parkin* single mutants, as well as in *Mtch Parkin* double mutants, compared to neighboring control enterocytes at 2 h APF (Fig 3G–3J). In addition, Mtch levels were elevated in *Parkin* RNAi knockdown cells compared to neighboring control enterocytes at 2 h APF (S5A, S5B Fig), and this is likely because mitochondrial clearance is inhibited in cells with reduced *Parkin* function. These data support a model with Mtch and Parkin functioning in the same genetic pathway to regulate mitophagy in developing enterocytes.

**Fig 4:**
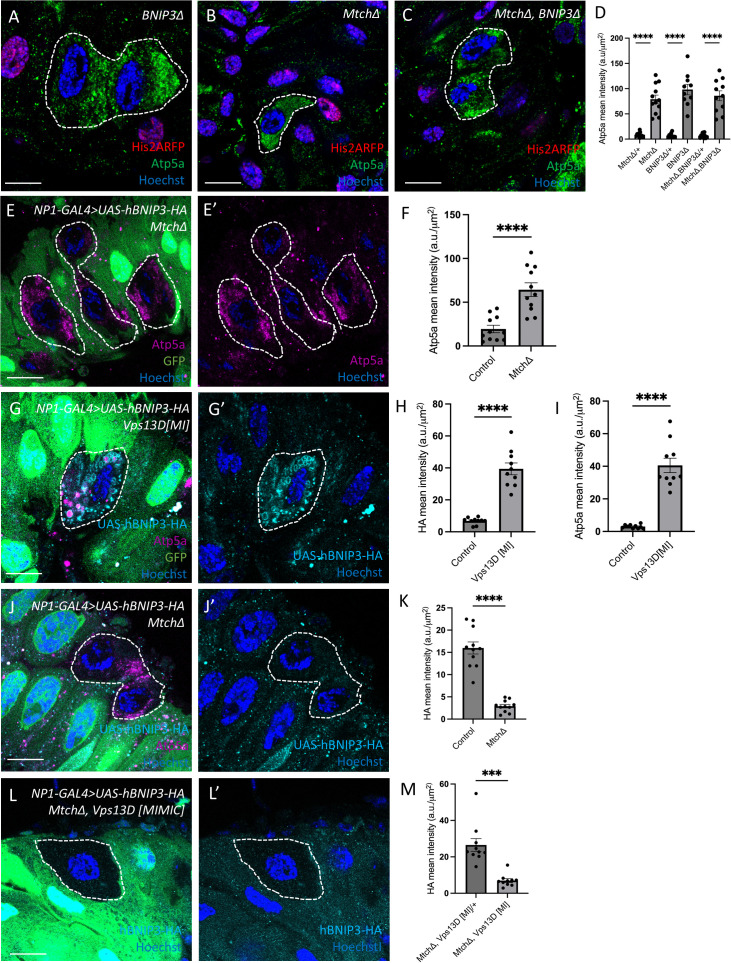
Mtch and BNIP3 function in a mitophagy pathway. **(A)** *BNIP3* mutant cells (absence of nuclear RFP labeled, dotted line) inhibit clearance of the mitochondrial Atp5a protein (green). Scale bar is 20 μm. **(B)** *Mtch* mutant cells (absence of nuclear RFP labeled, dotted line) inhibit clearance of the mitochondrial Atp5a protein (green). Scale bar is 20 μm. **(C)** *Mtch* and *BNIP3* double mutant cells (absence of nuclear RFP labeled, dotted line) inhibit clearance of the mitochondrial Atp5a protein (green). Scale bar is 20 μm. **(D)** Quantification of Atp5a mean fluorescence intensity in mutant vs. control cells (*n* = 10–12 mutant and *n* = 10–12 control cells across 3 independent animals were measured). Data are presented as mean ± SEM. ****p < 0.0001. (E and E’) Intestines expressing human BNIP3 (hBNIP3) in all enterocytes exhibit elevated levels of Atp5a (purple) in *Mtch* mutant cells (non-GFP labeled, dotted line) compared to neighboring control cells. Scale bar is 20 μm. **(F)** Quantification of Atp5a mean fluorescence intensity in *Mtch* mutant vs. control cells (*n* = 11 mutant and *n* = 11 control cells across 3 independent animals were measured). Data are presented as mean ± SEM. ****p < 0.0001. (G and G’) Intestine cells expressing human BNIP3 (hBNIP3, cyan) in all enterocytes exhibit elevated levels of Atp5a (purple) and rings of hBNIP3 in *Vps13D* mutant cells (non-GFP labeled, dotted line) compared to neighboring control cells indicating that hBNIP3 expression fails to rescue *Vps13D* mutant mitochondrial clearance. Scale bar is 20 μm. **(H)** Quantification of BNIP3-HA mean fluorescence intensity in *Vps13D* mutant vs. control cells (*n* = 10 mutant and *n* = 10 control cells across 3 independent animals were measured). Data are presented as mean ± SEM. n.s. = not significant. **(I)** Quantification of Atp5a mean fluorescence intensity in *Vps13D* mutant vs. control cells (*n* = 10 mutant and *n* = 10 control cells across 3 independent animals were measured). Data are presented as mean ± SEM. n.s. = not significant. (J and J’) Intestine cells expressing human BNIP3 (hBNIP3, cyan) in all enterocytes exhibit decreased levels of BNIP3-HA in *Mtch* mutant cells (non-GFP labeled, dotted line) compared to neighboring control cells. Scale bar is 20 μm. **(K)** Quantification of BNIP3-HA mean fluorescence intensity in *Mtch* mutant vs. control cells (*n* = 11 mutant and *n* = 11 control cells across 3 independent animals were measured). Data are presented as mean ± SEM. n.s. = not significant. (L and L’) Intestine cells expressing human BNIP3 (hBNIP3, cyan) in all enterocytes exhibit decreased levels of BNIP3-HA in *Mtch, Vps13D* double mutant cells (non-GFP labeled, dotted line) compared to neighboring control cells. Scale bar is 20 μm. **(M)** Quantification of BNIP3-HA mean fluorescence intensity in *Mtch*, Vps13D double mutants vs. control cells (*n* = 10 mutant and *n* = 10 control cells across 3 independent animals were measured). Data are presented as mean ± SEM. n.s. = not significant. The underlying data can be found in S1 Data.

BNIP3 encodes a mitophagy receptor that has been reported to function in a PINK1- and Parkin-dependent mitophagy pathway [18,19], and *BNIP3* is required for mitophagy in *Drosophila* enterocytes where PINK1 and Parkin regulate mitochondrial clearance [7]. This prompted us to investigate the relationship between Mtch and BNIP3 during intestine mitophagy. We compared single and double mutants to determine if Mtch and BNIP3 function in the same mitophagy pathway. These experiments revealed similar elevated levels of mitochondrial Atp5a in *Mtch* and *BNIP3* single and in *Mtch BNIP3* double mutants compared to neighboring control enterocytes at 2 h APF (Fig 4A–4D). These data indicate that *Mtch* and *BNIP3* function in the same genetic pathway to control enterocyte mitophagy during intestine development.

The Mtch orthologue MTCH2 has diverse functions in apoptosis, lipid metabolism and other processes [15,16,20]. MTCH2 has been reported to function as an insertase that is required for insertion of tail-anchored proteins into the mitochondrial outer membrane [17]. Since BNIP3 encodes a tail-anchored mitophagy receptor, we hypothesized that the function of Mtch in Vps13D-, PINK1-, and Parkin-dependent mitophagy may be through the proper regulation and localization of BNIP3 in the mitochondrial membrane. We utilized a strain of *Drosophila* that contains a stable human BNIP3 (hBNIP3) with an HA tagged transgene to test this hypothesis. We first expressed hBNIP3 in *Drosophila* intestine enterocytes, and observed similar low levels of Atp5a in *BNIP3* mutant and neighboring control cells (S6A, S6B Fig), indicating that hBNIP3 rescues mitophagy deficiency in *BNIP3* mutant enterocytes. By contrast, expression of hBNIP3 in *Mtch* mutant intestine enterocytes that lack green fluorescent protein (GFP) failed to rescue clearance of mitochondria based on elevated levels of mitochondrial Atp5a in *Mtch* mutant cells compared to lower levels of Atp5a in neighboring control cells (Fig 4E, 4F). In addition, we used immuno-staining of intestines with *Mtch*, *Vps13D,* and *Parkin* mutant cells that lack GFP at 2 h APF to interrogate expression and localization of hBNIP3. Although hBNIP3 exhibited rings of localization with mitochondria in *Vps13D* mutant cells (Fig 4G–4I), *Mtch* mutant enterocytes exhibited lower levels of diffuse hBNIP3 localization that was similar to neighboring control cells (Fig 4J, 4K). Similar to *Vps13D* mutant cells, *Parkin* mutant cells accumulated hBNIP3 and had elevated Atp5a (S6C, S6D Fig). Furthermore, when we expressed hBNIP3 in the intestines of *Mtch* and *Vps13D* double mutants, we saw decreased levels of hBNIP3, suggesting that Mtch is necessary for BNIP3 localization (Fig 4L, 4M). e additionally tested how hBNIP3 levels change in *Mtch* and *Parkin* double mutants and observed decreased levels of hBNIP3 (S6F, S6G Fig). These results indicate that Mtch is required for hBNIP3 localization to mitochondria and mitophagy, and suggest that the influence of Mtch on mitochondrial clearance is because of impaired tail-anchored protein insertion in mitochondria. We tested this possibility by investigating if *Mtch* loss influences the levels of the Hid and Zucchini (Zuc) mitochondrial tail-anchored proteins. Consistent with our hypothesis, *Mtch* mutant cells have reduced Hid and Zucchini levels compared to control neighboring cells (S6H–S6J Fig). Combined, these data indicate that Mtch functions in Vps13D-, PINK1-, and Parkin-dependent mitophagy, and that this is likely through the regulation of insertion of the tail-anchored protein and mitophagy receptor BNIP3.

## Discussion

Mitochondria are key organelles in cellular health that produce ATP as well as having other important functions, such as being signaling scaffolds and key sources of regulators of cell death [21]. Mitochondria are cleared by mitophagy to limit the release of inflammatory factors, including mitochondrial DNA and reactive oxygen species (ROS) [22]. Mitophagy selectively targets and eliminates damaged mitochondria before they can rupture and activate intrinsic apoptosis [23]. *Pink1* and *Parkin* are two of the most studied regulators of mitophagy [24], but alternative *Pink1-* and *Parkin-*independent mitophagy pathways exist [6,25]. Thus, it is important to identify additional factors that function in both *Pink1/Parkin-*dependent and -independent mitophagy pathways.

Using a developmental model to study mitophagy in a physiological setting [26], we discovered that *Mtch* is required for mitochondrial clearance in the *Drosophila* intestine. We screened for genes encoding proteins known to interact with Vps13D, a regulator of mitochondrial clearance in the *Drosophila* intestine, to gain insight into the function of Vps13D during mitophagy. CRISPR-targeted deletion of *Mtch* revealed strong phenotypes that are similar to *Vps13D* mutant cells. In addition, Vps13D protein levels are reduced in *Mtch* mutant cells. Single and double mutant analyses indicate that *Mtch* and *Vps13D* function in the same mitochondrial clearance pathway.

PINK1 and Parkin are widely studied because of their key roles in mitochondrial quality control and clearance [5]. We analyzed how Mtch and these two key mitophagy genes influence one another. Interestingly, PINK1 levels were significantly increased in *Mtch* mutants compared to control cells. PINK1 functions as a kinase to phosphorylate ubiquitin [5], and we observed significant increases in phosphorylated ubiquitin in *Mtch* cells. These data suggest that PINK1 function is activated in *Mtch* mutant cells despite a block in mitochondrial clearance by mitophagy. We also observed a dramatic increase in Mtch levels in *Pink1* mutants, but this is likely because of the failure of *Pink1* mutants to clear mitochondria. Our data also indicate that Mtch and Parkin function in the same mitophagy pathway, as double mutants exhibited similar increases in mitochondria compared to either *Parkin* or *Mtch* single mutant intestine cells. Thus, these studies indicate that Mtch functions downstream of Pink1 and Parkin in the regulation of mitophagy in intestine cells.

Mtch/MTCH2 has been reported to possess a variety of functions in diverse taxa, including lipid homeostasis [27], sperm development [28], stem cell maintenance [29,30], mitochondrial fusion [31], apoptosis [15], and most recently as a mitochondrial protein insertase [17]. *BNIP3* is required for mitophagy in the *Drosophila* intestine, and expression of hBNIP3 rescued the *BNIP3* mutant mitochondrial clearance defect in intestine enterocytes. In addition, single and double mutant analyses of *Mtch* and *Bnip3* mutants showed similar levels of elevated mitochondrial Atp5a, further indicating that these genes function in a common mitochondrial clearance genetic pathway. Since BNIP3 encodes a mitochondrial tail-anchored protein that acts as a mitophagy receptor [32], we tested the hypothesis that *Mtch* is required for proper BNIP3 expression and localization with mitochondria. Significantly, hBNIP3 could not rescue clearance of mitochondria in *Mtch* mutant intestine cells, and hBNIP3 levels were reduced in *Mtch* mutant cells compared to either *Vps13D* or *Parkin* mutant cells. Double mutants of both *Mtch* and *Vps13D* as well as *Mtch* and *Parkin* both showed reduced levels, which suggests that *Mtch* is essential for mitophagy despite BNIP3 levels being increased in single mutants of *Vps13D* and *Parkin*. In addition, the tail-anchored Mito-QC mitophagy sensor, as well as the mitochondrial tail-anchored proteins Hid and Zucchini, levels were also significantly decreased in *Mtch* mutant cells. By contrast and similar to Atp5a and Pink1, mitochondrial COX4 protein is increased in *Mtch* mutant cells, as are levels of TMRE, a reporter of mitochondrial function. Although we have not tested if mitochondrial clearance can be rescued by artificially tethering BNIP3 to mitochondria in *Mtch* mutant cells, our combined data suggest that BNIP3 mitochondrial localization is necessary for Mtch-mediated mitophagy. Furthermore, our data suggest that the mitochondrial insertase function of Mtch/MTCH2 may explain the broad range of cellular functions attributed to this important mitochondrial protein.

## Materials and methods

### Fly stocks

*Drosophila* strains used in this study are listed in the S2 Table with complete genotypes for each experiment in the S3 Table. All crosses were raised at 25 °C. The *w*^*1118*^ wild-type strain was used as control for TEM experiments. Flies were raised on standard cornmeal/molasses/agar media.

### Gene editing for deletion of Mtch and tagging of Mtch

The *MtchΔ* and *Mtch-2xHA* strains were edited using CRISPR/Cas9 technology [33]. The gRNAs for each strain were cloned into *Drosophila*-optimized U6b plasmids. For the *MtchΔ* strain, 0.8kb homology arms were synthesized by IDT and cloned into a TOPO TA vector. For the *Mtch-2xHA* strain, 1 kb homology arms were synthesized and cloned into a TOPO TA vector. A linker peptide was used between Mtch and the 2xHA peptide, and was designed to be on the C-terminus of Mtch. Plasmid DNA for gRNAs and template were injected into a vasa-cas9 *Drosophila* germline by Rainbow Genetics. Genotyping was done using primers outside of gRNAs for the *MtchΔ* strain, while genotyping was done for *Mtch-2xHA* using primers that included part of the *HA* sequence and *Mtch* sequence. Results were then sequenced (Azenta) to confirm the appropriate *MtchΔ* and *Mtch-2xHA* sequences. Sequences and gene blocks used are listed in S4 Table.

### Induction of mutant and RNAi-directed mosaic cell clones and whole intestine RNAi expression

Mosaic mutant cells and RNAi expression clones were induced as previously described [9]. For RNAi experiments, the RNAi of the gene of interest was crossed to *hs-flp;; ACT > CD2 > GAL4, UAS-DsRed* line to induce gene knockdown cells. Mutant cell clones (*Mtch, Vps13D, Parkin, BNIP3,* and respective double mutant combinations) were induced when crossed to a *hs-flp;; His2a-RFP, FRT2A* strain so that mutant cells were marked by the lack of RFP in the nucleus. For hBNIP3-HA experiments, a *hs-flp; NP1-GAL4; Ub-GFP, FRT2A* strain was used to induce mutant cell clones. For *PINK1[B9]* analysis, a *hs-flp, FRT19A;; Ub-RFP* strain was used to mark mutant cells by the lack of red fluorescence. NP1-GAL4 is an enterocyte-specific GAL4 driver that was used for TEM experiments and to express hBNIP3 and PINK1 RNAi in enterocytes of the entire intestine.

### Immunolabeling and microscopy

Crosses were kept in vials with media to mate for 5 h before being subjected to a 1-h heat shock at 37 °C to induce FLP/FRT recombination early in development. Animals were then collected as white prepupae, aged for 2 h at 25 °C on a PBS-soaked Kimwipe, and then promptly dissected in PBS to isolate the intestine. The intestines were then fixed in 4% PFA for 30 min and then incubated in the primary antibodies Ref(2)p (1:200), Atp5a (1:1000), phospho-ubiquitin (1:500), HA (1:500), FLAG (1:500), Atg8a (1:100), Vps13D (1:50), GFP (1:5000), and Hid (1:500). The next day the intestines were washed in 1x PBS 3 times for 15 min before being incubated in secondary antibodies (1:500 for all) for 2 h and Hoechst stained (1:200) for 30 min. Stained intestines were then transferred to microscope slides with Vectashield on them and mounted with a cover slip to be imaged within the next day. Images were taken on a Zeiss LSM700 and LSM900 using a 63x oil objective. At least three animals were imaged for each genotype, with a minimum of 10 cells analyzed per genotype per antibody stain.

For TMRE labeling and imaging, *Mtch* RNAi was crossed to *hsflp;P{y[+t7.7] w[+mC]=CoinFLP-GAL4}attP40 P{w[+mC]=UAS-2xEGFP}AH2* to induce *Mtch* knockdown cells marked by GFP. Animals were collected as white prepupae, aged for 2 h on a PBS-soaked Kimwipe, and promptly dissected in Schneider’s *Drosophila* Medium (Gibco, 21720024). The intestines were then incubated in the dark with 500 nM TMRE (Invitrogen, T669) diluted in Schneider’s medium at room temperature for 20 min. Intestines were immediately mounted in Schneider’s medium and imaged on a Zeiss LSM900 using a 63x oil objective. Each intestine was mounted separately, and one image was taken per intestine to reduce variability due to mechanical pressure on the intestine from mounting.

### RNA isolation and RT-qPCR analysis

Total RNA was extracted from 12 to 17 pooled intestines per replicate of staged white prepupae using RNeasy Plus Mini Kit (QIAGEN, 74134) and cDNA was synthesized by Super Script III First-Strand Kit (Invitrogen, 18080051) according to the manufacturer’s guidelines. Quantitative PCR (qPCR) was performed in triplicate with SYBR Green PCR Master Mix (Applied Biosystems, 4367659) using CFX96 Touch Real-Time PCR Detection System (Bio-Rad, CA, USA). The RNA levels of *Vps13D* were normalized to *RPL32* and are presented as means ± SEM. Primers sequences are listed in S4 Table.

### Transmission electron microscopy

Intestines were dissected from animals in PBS 2 h after pupariation. Intestines were then fixed overnight at 4 °C in 2.5% glutaraldehyde and 2% paraformaldehyde in 0.1 M sodium cacodylate buffer, pH 7.4. Tissue was then osmicated and washed in distilled water. Specimens were then stained en bloc in 1% aqueous uranyl acetate, dehydrated through a graded ethanol series, treated with propylene oxide and infiltrated in SIP-pon/Araldite for embedding. Ultrathin sections of the anterior region of the midgut of the intestine were collected and stained with uranyl acetate and lead citrate. At least 3 intestines were embedded and sectioned for analyses and quantification of each genotype. Images were collected using a Phillips CM10 TEM.

### Quantification and statistical analysis

Fiji Imaging software was used to analyze fluorescence intensity in all experiments. Statistical analyses were performed in Prism 10, and paired *t*-tests were used to analyze intensity differences between mutant and control cells. *P*-values were calculated based on *t* test values. No animals were excluded from these studies for any reason unless obvious damage occurred.

## Supporting information

S1 Fig *Mtch* RNAi knockdown cells accumulate markers of mitochondria and autophagy. **(A)** *Mtch* RNAi (v106996) knockdown cells (DsRed labeled, white dotted outline) accumulate the autophagy adaptor Ref(2)p (red) compared to control cells (non-DsRed labeled). Scale bar is 20 μm. (*n* = 6 *Mtch* and 6 control cells across 3 independent animals were measured). **(B)** Quantification of Ref(2)p fluorescence intensity in *Mtch* knockdown and control cells (*n* = 10 *Mtch* and *n* = 10 control cells across 3 independent animals were measured). ****p < 0.0001. **(C and C’)** *Mtch* RNAi knockdown cells (DsRed labeled, white dotted outline) accumulate the autophagy protein Atg8a (cyan). Scale bar is 20 μm. **(D)** Quantification of Atg8a fluorescence intensity in *Mtch* knockdown and control cells (*n* = 10 *Mtch* and *n* = 10 control cells across 3 independent animals were measured). ****p < 0.0001. **(E and E’)** *Mtch* RNAi (BL38986) knockdown cells (DsRed labeled, white dotted outline) accumulate the mitochondrial protein, Atp5 (cyan) compared to control cells (non-DsRed labeled). Scale bar is 20 μm. (*n* = 6 *Mtch* and 6 control cells across 3 independent animals were measured). **(F and F’)** *Mtch* RNAi (BL38986) knockdown cells (DsRed labeled, white dotted outline) accumulate the autophagy adaptor Ref(2)p (red) compared to control cells (non-DsRed labeled). Scale bar is 20 μm. (*n* = 6 *Mtch* and 6 control cells across 3 independent animals were measured). **(G and G’)** *Mtch* mutant cells (nonnuclear RFP, white dotted outline) accumulate the autophagy protein Atg8a (yellow). Scale bar is 20 μm. **(H)** Quantification of Atg8a fluorescence intensity in mutant and control cells (*n* = 10 *Mtch* mutant and *n* = 10 control cells across 3 independent animals were measured). Data are presented as mean ± SEM. ****p < 0.0001. The underlying data can be found in S1 Data.
(TIF)

S2 Fig *Mtch* cells have reduced mitochondrial protein reporters but do not influence cleaved caspase-3 levels. **(A–A’’)** Mito-QC was expressed in all intestinal enterocytes. Neither GFP nor RFP was detected in *Mtch* mutant cells (absence of nuclear RFP, white dotted outline) despite saturation of GFP and RFP fluorescence of the Mito-QC mitophagy sensor in neighboring control cells. (B) Electron micrograph of *w*^*1118*^ control enterocyte in the *Drosophila* intestine 2 h after puparium formation. Scale bar is 0.5 μm. **(B and B’)** *Mtch* mutant cells (nonnuclear RFP labeled, dotted line) have decreased mito-GFP (green) levels compared to control cells. Scale bar is 20 μm. (*n* = 6 *Mtch* mutant and 6 control cells across 2 independent animals were measured). **(C and C’)** *Mtch* mutant cells (nonnuclear RFP labeled, dotted line) have decreased COX4 (red) levels compared to control cells. Scale bar is 20 μm. **(D)** Quantification of COX4 fluorescence intensity in *Mtch* mutant and control cells (*n* = 31 knockdown and 31 control cells across 10 independent animals were measured). **p < 0.01. **(E and E’)** *Mtch* RNAi knockdown cells (GFP labeled, white dotted outline) accumulate TMRE (red) compared to control cells (non-GFP labeled). Scale bar is 20 μm. **(F)** Quantification of TMRE fluorescence intensity in *Mtch* RNAi knockdown cells and control cells (*n* = 9 knockdown and 9 control cells across 7 independent animals were measured). **p < 0.01. **(G and G’)** *Mtch* mutant cells (nonnuclear RFP labeled, dotted line) do not influence cleaved caspase-3 (purple) compared to control cells (nonnuclear RFP labeled). Scale bar is 20 μm. (*n* = 6 *Mtch* mutant and 6 control cells across 2 independent animals were measured). The underlying data can be found in S1 Data.
(TIF)

S3 Fig Mtch localizes with mitochondria and *Mtch* RNAi decreases Mtch-HA levels. **(A)** Diagram of 2x-HA tagged Mtch on the C-terminus, with a 15 amino acid linker between gene and epitope. **(B)** Merged image in control (*w*^*1118*^) showing co-localization between Mtch-2xHA (red) and mitochondrial protein Atp5a (green). Scale bar is 20 μm. **(B’)** Mtch-2xHA staining in *w*^*1118*^ developing intestines (red). **(B’’)** Mitochondrial protein Atp5a (green) staining in *w*^*1118*^ developing intestines. **(C and C’)** *Mtch* RNAi (v106996) knockdown cells (DsRed labeled, dotted line) stained with HA show decreased Mtch-2xHA as a result of RNAi knockdown compared to neighboring control cells (non-DsRed labeled). Scale bar is 20 μm. **(D)** Quantification of Mtch-2xHA fluorescence intensity in *Mtch* knockdown and control cells (*n* = 10 knockdown and 10 control cells across 3 independent animals were measured). ***p < 0.001. **(E)** Wild-type intestine enterocyte at 2 h APF stained to detect Vps13D-3xFLAG (green) and Mtch-2xHA (purple). Scale bar is 20 μm. **(F** and **G)** Enlargements from (E) in two separate areas (dotted square) showing juxtaposed and overlapping staining of Vps13D and Mtch (arrows). **(H)** Quantitative RT-PCR analysis of *Vps13D* RNA in Intestines that express *Mtch* RNAi in enterocytes. Total RNA was extracted from 12 to 17 pooled intestines per replicate of staged white prepupae. Equal amounts of RNA isolated from control (NP1-GAL4/UAS-*GFP*) and experimental (NP1-GAL4/UAS-*Mtch* RNAi) were analyzed by quantitative RT-PCR. Quantification of *Vps13D* RNA levels in control and *Mtch* RNAi intestines (*n* = 3 control and *Mtch* RNAi biological replicates). Data are presented as mean ± SEM. ns = not significant. The underlying data can be found in S1 Data.
(TIF)

S4 Fig *Mtch* and *Vps13D* have similar influence on Atg8a. **(A and A’)** *Mtch* mutant cells (absence of nuclear RFP labeled, dotted line) inhibit clearance of autophagy protein Atg8a (yellow) compared to neighboring controls (nuclear RFP labeled). Scale bar is 20 μm. **(B and B’)** *Vps13D* mutant cells (absence of nuclear RFP labeled, white dotted outline) inhibit clearance of autophagy protein Atg8a (yellow) compared to neighboring controls (nuclear RFP labeled). Scale bar is 20 μm. **(C and C’)** *Mtch Vps13D* double mutant cells (absence of nuclear RFP labeled, dotted line) inhibit clearance of autophagy protein Atg8a (yellow) compared to neighboring controls (nuclear RFP labeled). Scale bar is 20 μm. **(D)** Quantification of Atg8a mean fluorescence intensity in *Mtch, Vps13D*, and *Mtch Vps13D* mutant cells (*n* = 10 for each single and double mutant, and *n* = 10 control cells across 3 independent animals were measured). Data are presented as mean ± SEM. ***p < 0.001, ****p < 0.0001. The underlying data can be found in S1 Data.
(TIF)

S5 Fig Parkin knockdown cells possess increased Mtch levels. **(A and A’)** *Parkin* RNAi knockdown cells (DsRed labeled, dotted line) exhibit increased levels of Mtch-2xHA (purple) compared to control cells (non-DsRed labeled). Scale bar is 20 μm. **(B)** Quantification of Mtch-2xHA mean fluorescence intensity in mutant versus control cells (*n* = 10 knockdown and *n* = 10 control cells across 3 independent animals were measured). Data are presented as mean ± SEM. ***p < 0.001, ****p < 0.0001. The underlying data can be found in S1 Data.
(TIF)

S6 Fig hBNIP3 expression rescues *BNIP3* mutant clearance of mitochondria, and *Parkin* mutant mitochondrial clearance defects are not rescued by expression of hBNIP3. **(A and A’)** Intestine cells expressing human BNIP3 (hBNIP3) in *BNIP3* mutant cells (non-GFP labeled, dotted line) show similar levels of Atp5a clearance compared to control. (*n* = 10 knockout and 10 control cells across 3 independent animals were measured). Scale bar is 20 μm. **(B)** Quantification of Atp5a mean fluorescence intensity in *Mtch* mutant versus control cells (*n* = 10 mutant and *n* = 10 control cells across 3 independent animals were measured). Data are presented as mean ± SEM. n.s. = not significant. **(C and C’)** Intestine cells expressing hBNIP3 in *Parkin* mutant cells (non-GFP labeled, dotted line) exhibit elevated levels of Atp5a clearance compared to control. Scale bar is 20 μm. **(D)** Quantification of Atp5a mean fluorescence intensity in *Parkin* mutant versus control cells (*n* = 10 mutant and *n* = 10 control cells across 3 independent animals were measured). Data are presented as mean ± SEM. n.s. = not significant ****p < 0.0001. **(E)** Quantification of hBNIP3-HA mean fluorescence intensity in *Parkin* mutant versus control cells (*n* = 10 mutant and *n* = 10 control cells across 3 independent animals were measured). Data are presented as mean ± SEM. n.s. = not significant. ****p < 0.0001. **(F and F’)** Intestine cells expressing hBNIP3 in *Mtch, Parkin* double mutant cells (non-GFP labeled, dotted line) exhibit elevated levels of Atp5a clearance compared to control. Scale bar is 20 μm. **(G)** Quantification of hBNIP3-HA mean fluorescence intensity in *Mtch, Parkin* double mutant versus control cells (*n* = 10 mutant and *n* = 10 control cells across 3 independent animals were measured). Data are presented as mean ± SEM. n.s. = not significant. ****p < 0.0001. **(H–H”)** *Mtch* mutant cells (absence of nuclear RFP labeled, dotted line) possess reduced levels of the tail-anchored mitochondrial proteins Hid (magenta) and Zuc (green) compared to neighboring controls (nuclear RFP labeled). Scale bar is 20 μm. **(I)** Quantification of Hid mean fluorescence intensity in *Mtch* mutant cells (*n* = 10 for mutant and *n* = 10 control cells across 3 independent animals were measured). Data are presented as mean ± SEM. ****p < 0.0001. **(J)** Quantification of Zuc mean fluorescence intensity in *Mtch* mutant cells (*n* = 10 for mutant and *n* = 10 control cells across 3 independent animals were measured). Data are presented as mean ± SEM. ****p < 0.0001. The underlying data can be found in S1 Data.
(TIF)

S1 Table Screen for genes encoding Vps13D interacting proteins that influence mitochondrial clearance. (DOCX)

S2 Table *Drosophila* strains. (DOCX)

S3 Table Genotypes of *Drosophila* strains by figure. (DOCX)

S4 Table Oligonucleotides and gBlocks. (DOCX)

S1 Data (XLSX)

## Abbreviations

ER: endoplasmic reticulum
GFP: green fluorescent protein
hBNIP3: human BNIP3
mitophagy: mitochondrial-selective autophagy
qPCR: Quantitative PCR
RFP: red fluorescent protein
ROS: reactive oxygen species
TEM: Transmission Electron Microscopy
