# Supplementary figures and images for: The conserved outer mitochondrial membrane protein Mtch regulates mitophagy during Drosophila intestinal development
Source: PLoS Biol. 2026 Jan 23;24(1):e3003616. doi: 10.1371/journal.pbio.3003616 (PMC12829841; doi:10.1371/journal.pbio.3003616)

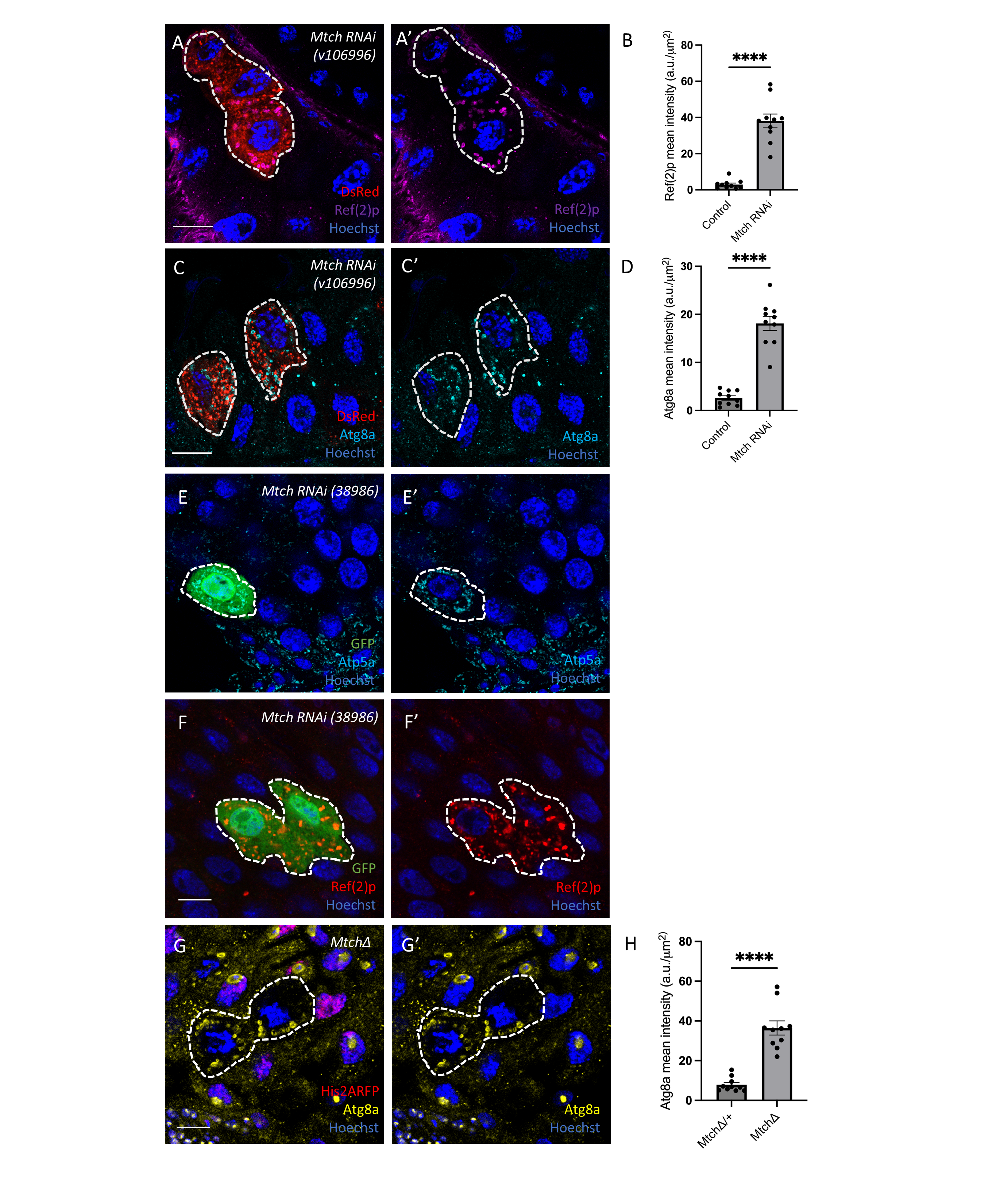

Supplement: S1 Fig — (A) Mtch RNAi (v106996) knockdown cells (DsRed labeled, white dotted outline) accumulate the autophagy adaptor Ref(2)p (red) compared to control cells (non-DsRed labeled). Scale bar is 20 μm. (n = 6 Mtch and 6 control cells across 3 independent animals were measured). (B) Quantification of Ref(2)p fluorescence intensity in Mtch knockdown and control cells (n = 10 Mtch and n = 10 control cells across 3 independent animals were measured). ****p < 0.0001. (C and C’) Mtch RNAi knockdown cells (DsRed labeled, white dotted outline) accumulate the autophagy protein Atg8a (cyan). Scale bar is 20 μm. (D) Quantification of Atg8a fluorescence intensity in Mtch knockdown and control cells (n = 10 Mtch and n = 10 control cells across 3 independent animals were measured). ****p < 0.0001. (E and E’) Mtch RNAi (BL38986) knockdown cells (DsRed labeled, white dotted outline) accumulate the mitochondrial protein, Atp5 (cyan) compared to control cells (non-DsRed labeled). Scale bar is 20 μm. (n = 6 Mtch and 6 control cells across 3 independent animals were measured). (F and F’) Mtch RNAi (BL38986) knockdown cells (DsRed labeled, white dotted outline) accumulate the autophagy adaptor Ref(2)p (red) compared to control cells (non-DsRed labeled). Scale bar is 20 μm. (n = 6 Mtch and 6 control cells across 3 independent animals were measured). (G and G’) Mtch mutant cells (nonnuclear RFP, white dotted outline) accumulate the autophagy protein Atg8a (yellow). Scale bar is 20 μm. (H) Quantification of Atg8a fluorescence intensity in mutant and control cells (n = 10 Mtch mutant and n = 10 control cells across 3 independent animals were measured). Data are presented as mean ± SEM. ****p < 0.0001. The underlying data can be found in S1 Data. (TIF) [file pbio.3003616.s001.tif]

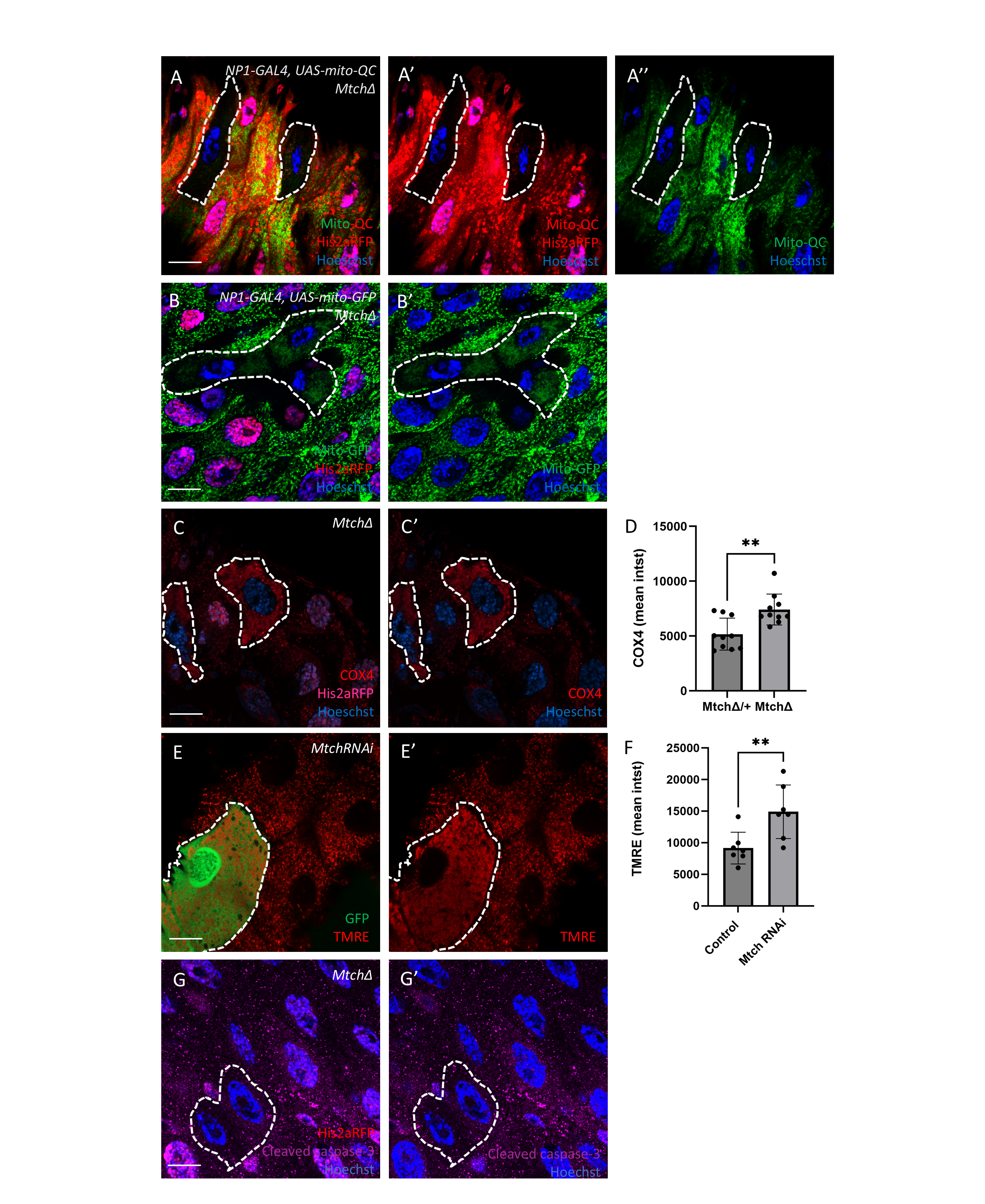

Supplement: S2 Fig — (A–A’’) Mito-QC was expressed in all intestinal enterocytes. Neither GFP nor RFP was detected in Mtch mutant cells (absence of nuclear RFP, white dotted outline) despite saturation of GFP and RFP fluorescence of the Mito-QC mitophagy sensor in neighboring control cells. (B) Electron micrograph of w1118 control enterocyte in the Drosophila intestine 2 h after puparium formation. Scale bar is 0.5 μm. (B and B’) Mtch mutant cells (nonnuclear RFP labeled, dotted line) have decreased mito-GFP (green) levels compared to control cells. Scale bar is 20 μm. (n = 6 Mtch mutant and 6 control cells across 2 independent animals were measured). (C and C’) Mtch mutant cells (nonnuclear RFP labeled, dotted line) have decreased COX4 (red) levels compared to control cells. Scale bar is 20 μm. (D) Quantification of COX4 fluorescence intensity in Mtch mutant and control cells (n = 31 knockdown and 31 control cells across 10 independent animals were measured). **p < 0.01. (E and E’) Mtch RNAi knockdown cells (GFP labeled, white dotted outline) accumulate TMRE (red) compared to control cells (non-GFP labeled). Scale bar is 20 μm. (F) Quantification of TMRE fluorescence intensity in Mtch RNAi knockdown cells and control cells (n = 9 knockdown and 9 control cells across 7 independent animals were measured). **p < 0.01. (G and G’) Mtch mutant cells (nonnuclear RFP labeled, dotted line) do not influence cleaved caspase-3 (purple) compared to control cells (nonnuclear RFP labeled). Scale bar is 20 μm. (n = 6 Mtch mutant and 6 control cells across 2 independent animals were measured). The underlying data can be found in S1 Data. (TIF) [file pbio.3003616.s002.tif]

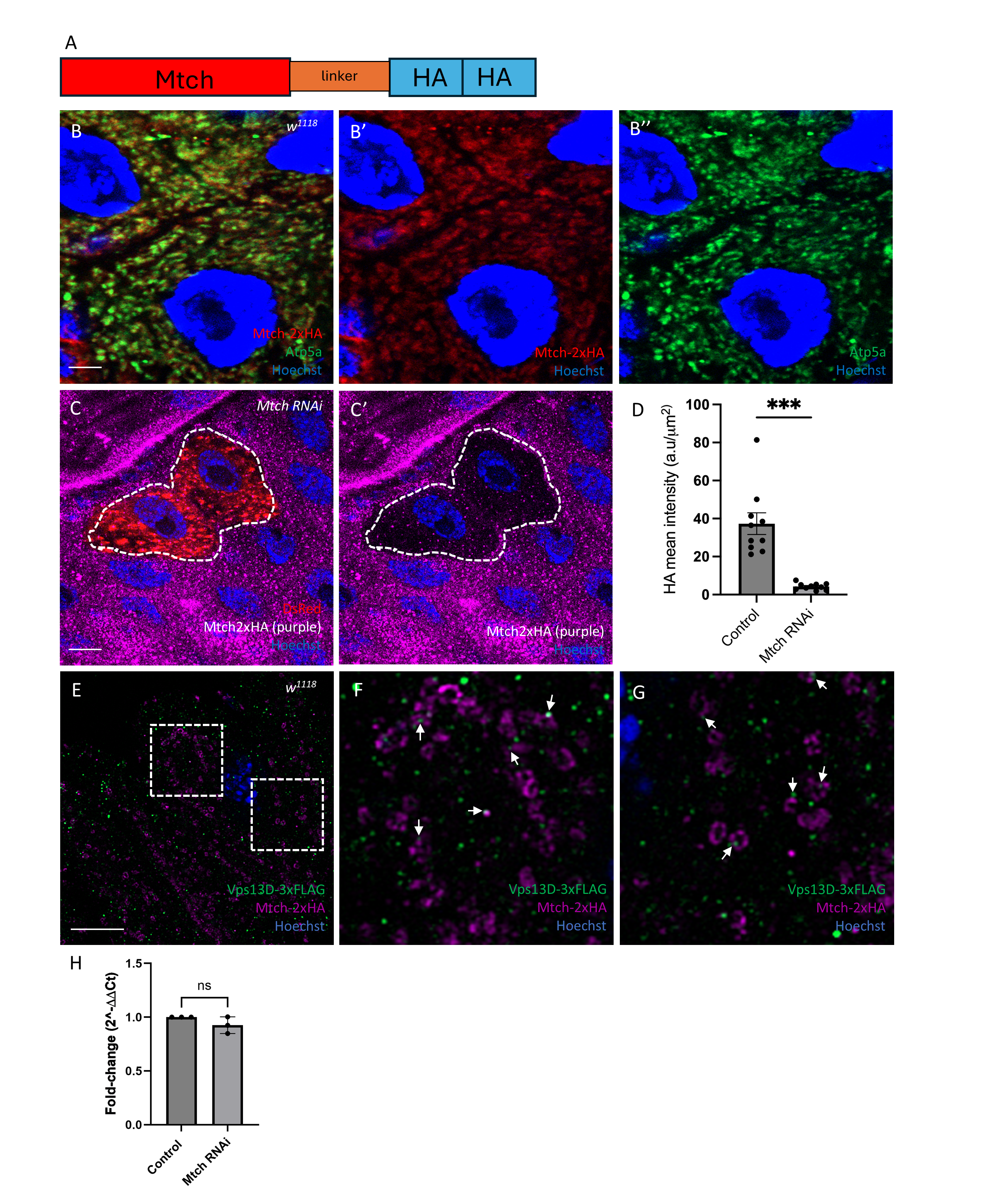

Supplement: S3 Fig — (A) Diagram of 2x-HA tagged Mtch on the C-terminus, with a 15 amino acid linker between gene and epitope. (B) Merged image in control (w1118) showing co-localization between Mtch-2xHA (red) and mitochondrial protein Atp5a (green). Scale bar is 20 μm. (B’) Mtch-2xHA staining in w1118 developing intestines (red). (B’’) Mitochondrial protein Atp5a (green) staining in w1118 developing intestines. (C and C’) Mtch RNAi (v106996) knockdown cells (DsRed labeled, dotted line) stained with HA show decreased Mtch-2xHA as a result of RNAi knockdown compared to neighboring control cells (non-DsRed labeled). Scale bar is 20 μm. (D) Quantification of Mtch-2xHA fluorescence intensity in Mtch knockdown and control cells (n = 10 knockdown and 10 control cells across 3 independent animals were measured). ***p < 0.001. (E) Wild-type intestine enterocyte at 2 h APF stained to detect Vps13D-3xFLAG (green) and Mtch-2xHA (purple). Scale bar is 20 μm. (F and G) Enlargements from (E) in two separate areas (dotted square) showing juxtaposed and overlapping staining of Vps13D and Mtch (arrows). (H) Quantitative RT-PCR analysis of Vps13D RNA in Intestines that express Mtch RNAi in enterocytes. Total RNA was extracted from 12 to 17 pooled intestines per replicate of staged white prepupae. Equal amounts of RNA isolated from control (NP1-GAL4/UAS-GFP) and experimental (NP1-GAL4/UAS-Mtch RNAi) were analyzed by quantitative RT-PCR. Quantification of Vps13D RNA levels in control and Mtch RNAi intestines (n = 3 control and Mtch RNAi biological replicates). Data are presented as mean ± SEM. ns = not significant. The underlying data can be found in S1 Data. (TIF) [file pbio.3003616.s003.tif]

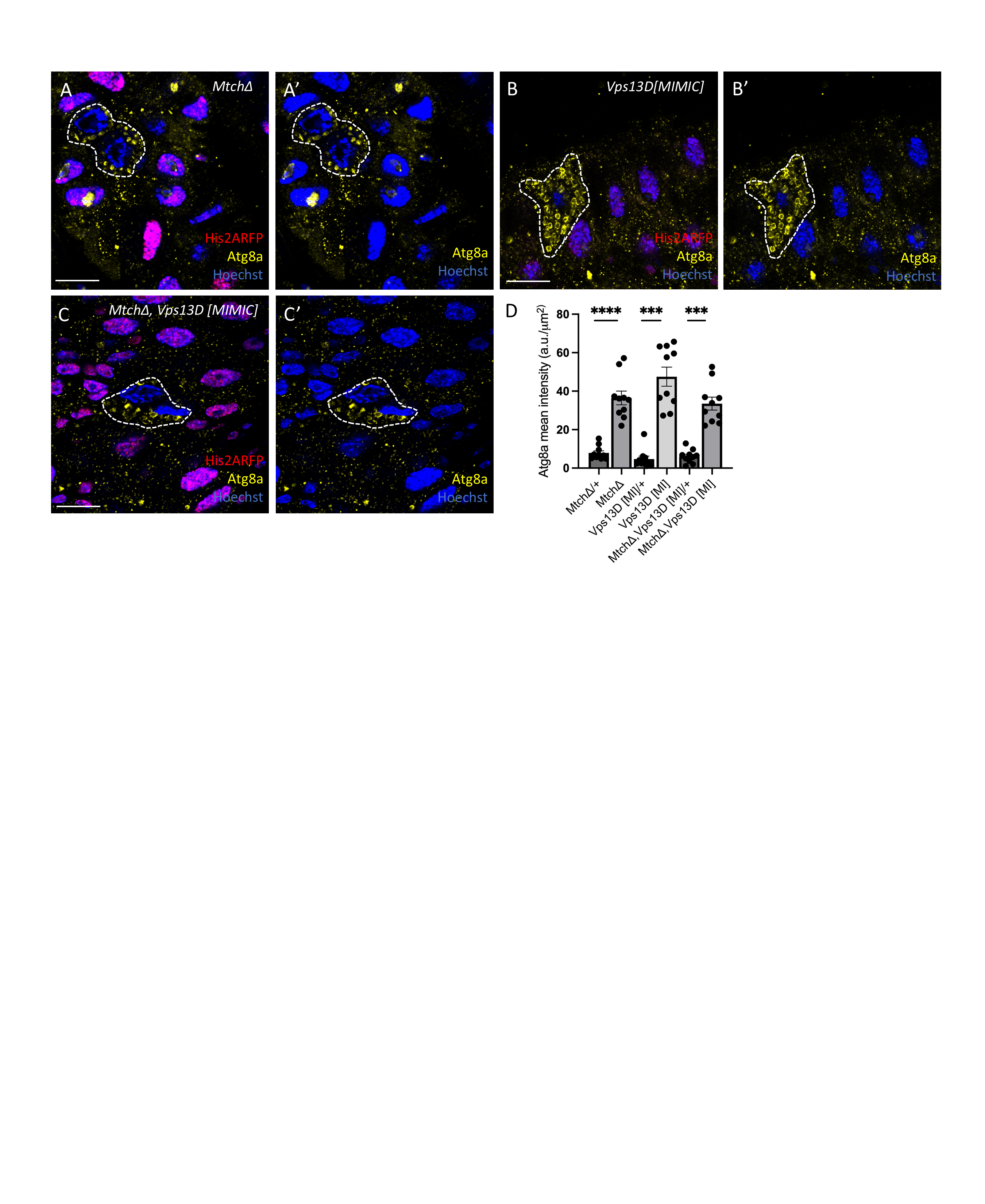

Supplement: S4 Fig — (A and A’) Mtch mutant cells (absence of nuclear RFP labeled, dotted line) inhibit clearance of autophagy protein Atg8a (yellow) compared to neighboring controls (nuclear RFP labeled). Scale bar is 20 μm. (B and B’) Vps13D mutant cells (absence of nuclear RFP labeled, white dotted outline) inhibit clearance of autophagy protein Atg8a (yellow) compared to neighboring controls (nuclear RFP labeled). Scale bar is 20 μm. (C and C’) Mtch Vps13D double mutant cells (absence of nuclear RFP labeled, dotted line) inhibit clearance of autophagy protein Atg8a (yellow) compared to neighboring controls (nuclear RFP labeled). Scale bar is 20 μm. (D) Quantification of Atg8a mean fluorescence intensity in Mtch, Vps13D, and Mtch Vps13D mutant cells (n = 10 for each single and double mutant, and n = 10 control cells across 3 independent animals were measured). Data are presented as mean ± SEM. ***p < 0.001, ****p < 0.0001. The underlying data can be found in S1 Data. (TIF) [file pbio.3003616.s004.tif]

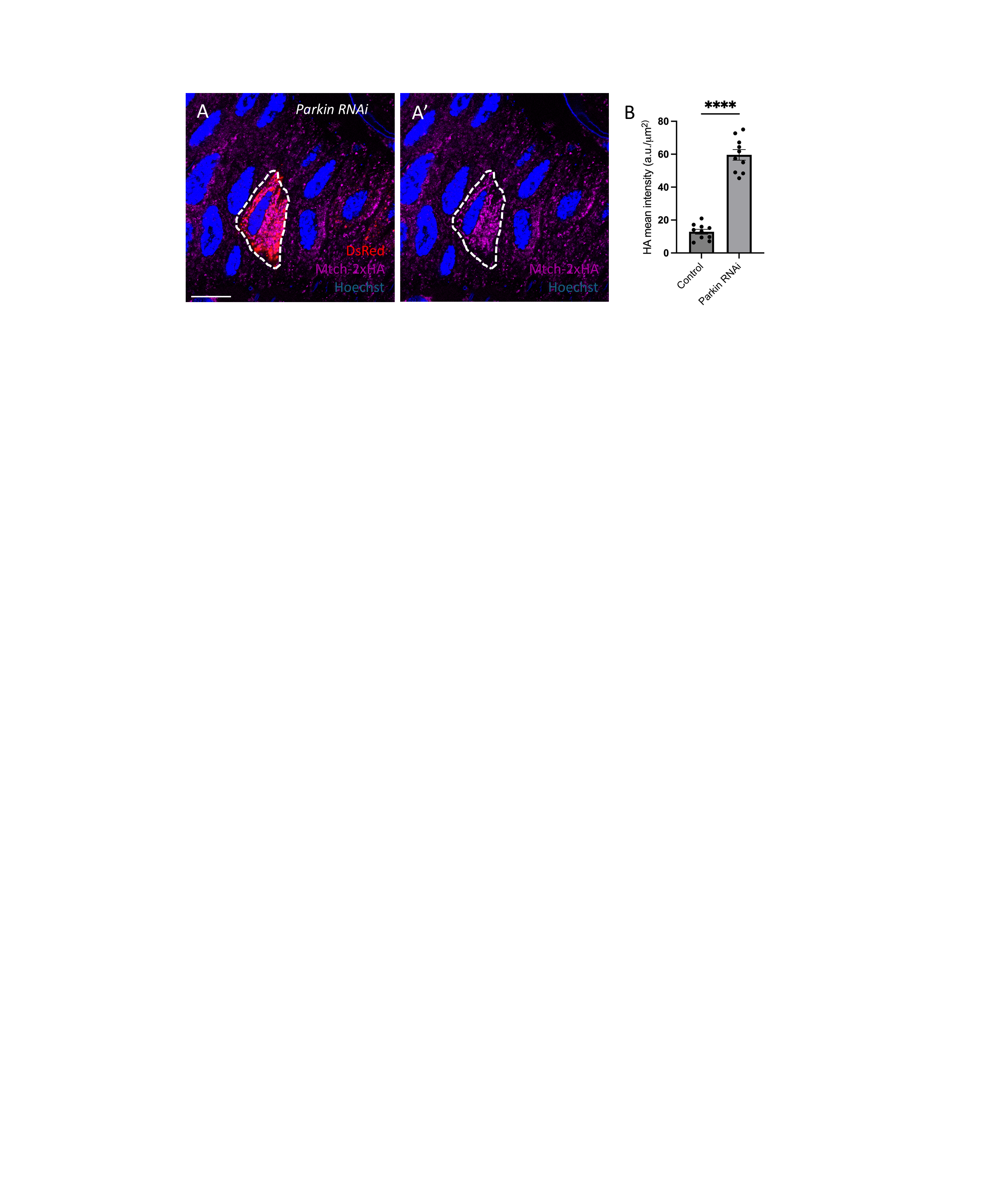

Supplement: S5 Fig — (A and A’) Parkin RNAi knockdown cells (DsRed labeled, dotted line) exhibit increased levels of Mtch-2xHA (purple) compared to control cells (non-DsRed labeled). Scale bar is 20 μm. (B) Quantification of Mtch-2xHA mean fluorescence intensity in mutant versus control cells (n = 10 knockdown and n = 10 control cells across 3 independent animals were measured). Data are presented as mean ± SEM. ***p < 0.001, ****p < 0.0001. The underlying data can be found in S1 Data. (TIF) [file pbio.3003616.s005.tif]

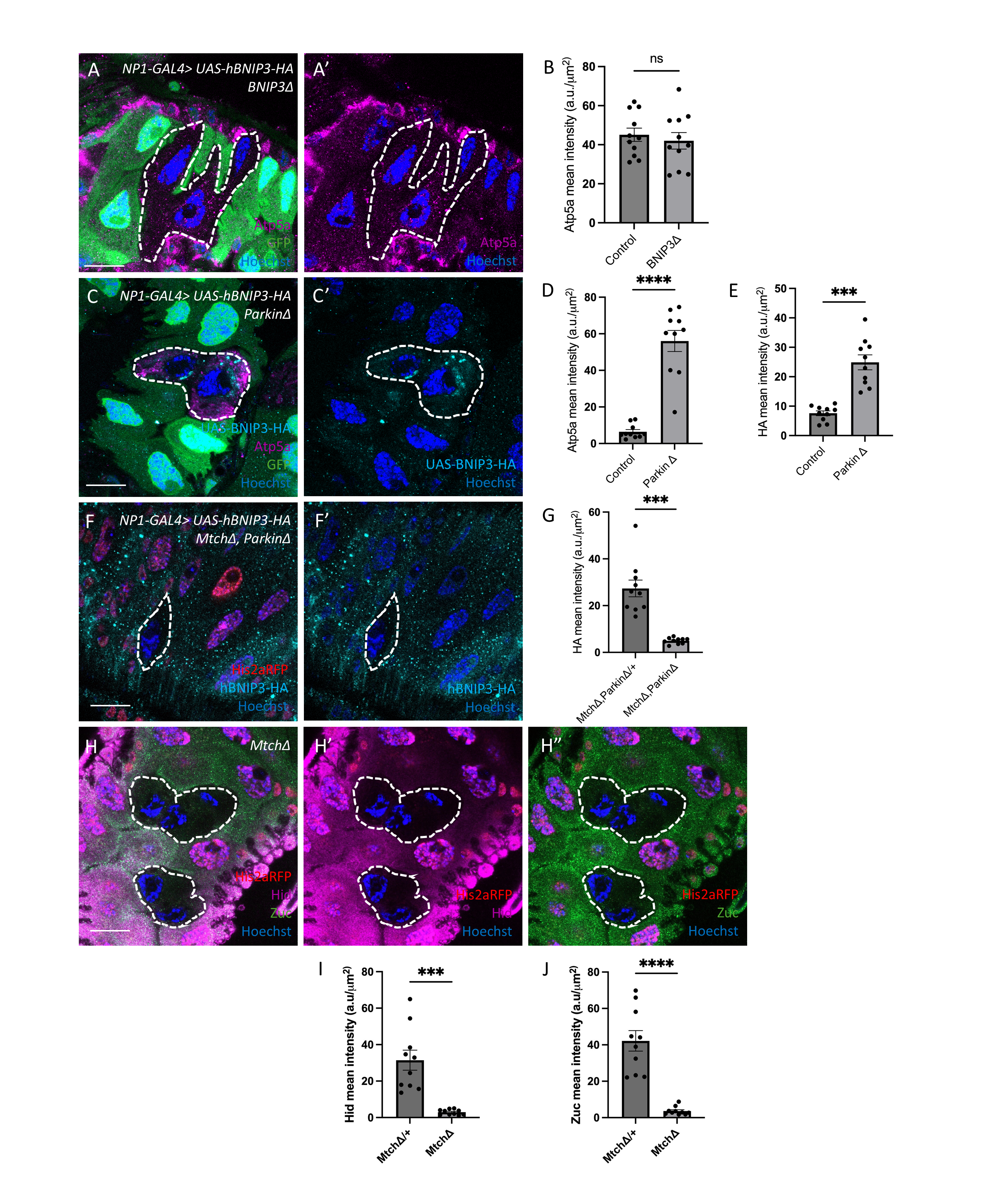

Supplement: S6 Fig — (A and A’) Intestine cells expressing human BNIP3 (hBNIP3) in BNIP3 mutant cells (non-GFP labeled, dotted line) show similar levels of Atp5a clearance compared to control. (n = 10 knockout and 10 control cells across 3 independent animals were measured). Scale bar is 20 μm. (B) Quantification of Atp5a mean fluorescence intensity in Mtch mutant versus control cells (n = 10 mutant and n = 10 control cells across 3 independent animals were measured). Data are presented as mean ± SEM. n.s. = not significant. (C and C’) Intestine cells expressing hBNIP3 in Parkin mutant cells (non-GFP labeled, dotted line) exhibit elevated levels of Atp5a clearance compared to control. Scale bar is 20 μm. (D) Quantification of Atp5a mean fluorescence intensity in Parkin mutant versus control cells (n = 10 mutant and n = 10 control cells across 3 independent animals were measured). Data are presented as mean ± SEM. n.s. = not significant ****p < 0.0001. (E) Quantification of hBNIP3-HA mean fluorescence intensity in Parkin mutant versus control cells (n = 10 mutant and n = 10 control cells across 3 independent animals were measured). Data are presented as mean ± SEM. n.s. = not significant. ****p < 0.0001. (F and F’) Intestine cells expressing hBNIP3 in Mtch, Parkin double mutant cells (non-GFP labeled, dotted line) exhibit elevated levels of Atp5a clearance compared to control. Scale bar is 20 μm. (G) Quantification of hBNIP3-HA mean fluorescence intensity in Mtch, Parkin double mutant versus control cells (n = 10 mutant and n = 10 control cells across 3 independent animals were measured). Data are presented as mean ± SEM. n.s. = not significant. ****p < 0.0001. (H–H”) Mtch mutant cells (absence of nuclear RFP labeled, dotted line) possess reduced levels of the tail-anchored mitochondrial proteins Hid (magenta) and Zuc (green) compared to neighboring controls (nuclear RFP labeled). Scale bar is 20 μm. (I) Quantification of Hid mean fluorescence intensity in Mtch mutant cells (n = 10 for m [file pbio.3003616.s006.tif]
