# Supplementary material for: The conserved outer mitochondrial membrane protein Mtch regulates mitophagy during Drosophila intestinal development
Source: PLoS Biol. 2026 Jan 23;24(1):e3003616. doi: 10.1371/journal.pbio.3003616 (PMC12829841; doi:10.1371/journal.pbio.3003616)
Supplement: S1 Table — (DOCX) [file pbio.3003616.s007.docx]

**S1 Table.** Screen for genes encoding Vps13D interacting proteins that influence mitochondrial clearance.

| Human Protein | Fly Homolog | Mitochondrial clearance defect phenotype? |
| --- | --- | --- |
| MFN2 | Marf | yes |
| OCIAD1 | Asrij | no |
| RHOT2 | Miro | no |
| SLC25A46 | Slc25a46b | no |
| MTCH1 | CG10920 | no |
| FIS1 | Fis1 | yes |
| EXD2 | Exd2 | no |
| MTX2 | CG8004 | no |
| MTCH2 | Mtch | yes |
| MARCH5 | March5 | no |
| MAVS | none | Not conserved |
| RMDN3 | none | Not conserved |

Table S1
