# Supplementary material for: The conserved outer mitochondrial membrane protein Mtch regulates mitophagy during Drosophila intestinal development
Source: PLoS Biol. 2026 Jan 23;24(1):e3003616. doi: 10.1371/journal.pbio.3003616 (PMC12829841; doi:10.1371/journal.pbio.3003616)
Supplement: S2 Table — (DOCX) [file pbio.3003616.s008.docx]

| **S2 Table. *Drosophila* strains** | | |
| --- | --- | --- |
| *Mtch RNAi* | Vienna Stock Center | V106996 |
| *Mtch RNAi* | Bloomington Drosophila Stock Center | BL38986 |
| *MtchΔ* | This study | N/A |
| *PINK1-GFP* | Hong Xu | N/A |
| *PINK1 [B9]* | Jongkyeong Chung | N/A |
| *ParkΔ* | Eric Baehrecke | N/A |
| *BNIP3Δ* | Eric Baehrecke | N/A |
| *PINK1 RNAi* | Vienna Drosophila Stock Center | 109614 |
| *Parkin RNAi* | Bloomington Drosophila stock center | 104363 |
| *Vps13D RNAi* | Vienna Drosophila Stock Center | 41792 |
| *BNIP3 RNAi* | Bloomington Drosophila stock Center | 42494 |
| *Vps13D [MI11101]* | Bloomington Drosophila stock center | 56282 |
| *hsflp;; His2Av-mRFP, FRT2a* | Bloomington Drosophila stock center | 34498 |
| *hsflp, FRT19a;; Ub-mRFP* | Bloomington Drosophila stock center | 31416 |
| *hsflp;; Act(CD2)GAL4, UAS-dsred* | Eric Baehrecke | N/A |
| *Hsflp; NP1-GAL4; Ub-GFP, FRT2A* | Eric Baehrecke | N/A |
| *Vps13D (ΔUBA*) | Eric Baehrecke | N/A |
| *NP1-GAL4* | Eric Baehrecke | N/A |
| *Vasa-cas9* | Bloomington Drosophila stock Center | 56552 |
| *Vps13D-3xFLAG* | Eric Baehrecke | N/A |
| *Mtch-2xHA* | This study | N/A |
| *UAS-hBNIP3-HA* | Ruoxi Wang | N/A |
| *Parkin2xHA* | Ruoxi Wang | N/A |
