# Supplementary material for: The conserved outer mitochondrial membrane protein Mtch regulates mitophagy during Drosophila intestinal development
Source: PLoS Biol. 2026 Jan 23;24(1):e3003616. doi: 10.1371/journal.pbio.3003616 (PMC12829841; doi:10.1371/journal.pbio.3003616)
Supplement: S3 Table — (DOCX) [file pbio.3003616.s009.docx]

| **S3 Table. Genotypes of *Drosophila* strains by figure** |
| --- |

| **Figure** | **Genotype** |
| --- | --- |
| **Fig 1** |  |
| A,A’ | hs-flp; Mtch-IR (v106696); Actin (CD2) GAL4, UAS-DsRed |
| C,C’ | hs-flp;; His2Av-mRFP, FRT2A*/ MtchΔ* FRT2A |
| E,E’ | hs-flp;; His2Av-mRFP, FRT2A*/ MtchΔ* FRT2A |
| G | +;+;+;+ |
| H | +;NP1-GAL4; Mtch-IR (v106696);+ |
|  |  |
| **Fig 2** |  |
| A, A’ | hs-flp; His2Av-mRFP, FRT2A*/ MtchΔ* FRT2A |
| C | hs-flp; Vps13D-IR (BL41792); Actin (CD2) GAL4, UAS-DsRed |
| D | hs-flp; Vps13D-IR (BL41792);+;+ |
| D’ | hs-flp; Vps13D-IR (BL41792); Actin (CD2) GAL4, UAS- DsRed |
| F,F’ | hs-flp; His2Av-mRFP, FRT2A*/ MtchΔ* FRT2A |
| G,G’ | hs-flp;; His2Av-mRFP, FRT2A*/ Vps13D [MI11101]* FRT2A |
| H,H’ | hs-flp;; His2Av-mRFP, FRT2A*/ MtchΔ,Vps13D [MI11101]* FRT2A |
|  |  |
| **Fig 3** |  |
| A,A’ | hs-flp, PINK-GFP;; His2Av-mRFP, FRT2A*/ MtchΔ* FRT2A |
| C,C’ | hs-flp;; His2Av-mRFP, FRT2A*/ MtchΔ* FRT2A |
| E,E’ | Hs-flp, PINK1[B9], FRT19A (males only) |
| G | hs-flp;; His2Av-mRFP, FRT2A*/ ParkΔ* FRT2A |
| H | hs-flp;; His2Av-mRFP, FRT2A*/ MtchΔ* FRT2A |
| I | hs-flp;; His2Av-mRFP, FRT2A*/ ParkΔ ,MtchΔ* FRT2A |
|  |  |
| **Fig 4** |  |
| A | hs-flp;; His2Av-mRFP, FRT2A*/ BNIP3Δ* FRT2A |
| B | hs-flp;; His2Av-mRFP, FRT2A*/ MtchΔ* FRT2A |
| C | hs-flp;; His2Av-mRFP, FRT2A*/ BNIP3Δ ,MtchΔ* FRT2A |
| E,E’ | hs-flp; NP1-GAL4/UAS-hBNIP3-HA; Ub-GFP FRT2A/ *MtchΔ* FRT2A |
| G,G’ | hs-flp; NP1-GAL4/UAS-hBNIP3-HA; Ub-GFP FRT2A/ *Vps13D [MI11101*] FRT2A |
| J, J’ | hs-flp; NP1-GAL4/UAS-hBNIP3-HA; Ub-GFP FRT2A/ *MtchΔ* FRT2A |
| L,L’ | hs-flp; NP1-GAL4/UAS-hBNIP3-HA; Ub-GFP FRT2A/ *Vps13D [MI11101] ,MtchΔ* FRT2A |
|  |  |
| **Fig S1** |  |
| A, A’ | hs-flp; Mtch-IR (v106696); Actin (CD2) GAL4, UAS-DsRed |
| C,C’ | hs-flp; Mtch-IR (v106696); Actin (CD2) GAL4, UAS-DsRed |
| E,E’ | hs-flp;; Actin (CD2) GAL4, UAS-GFP/ Mtch-IR (BL38986) |
| F,F’ | hs-flp;; Actin (CD2) GAL4, UAS-GFP/ Mtch-IR (BL38986) |
| G,G’ | hs-flp;; His2Av-mRFP, FRT2A*/ MtchΔ* FRT2A |
|  |  |
| **Fig S2** |  |
| A,A’,A’’ | Hs-flp;Np1-GAL4, UAS-mito-QC; *His2Av-mRFP,* FRT2A*/ MtchΔ* FRT2A |
| B,B’ | Hs-flp;Np1-GAL4, UAS-mito-GFP; *His2Av-mRFP,* FRT2A*/ MtchΔ* FRT2A |
| C,C’ | hs-flp;; His2Av-mRFP, FRT2A*/ MtchΔ* FRT2A |
| E,E’ | hs-flp;; Actin (CD2) GAL4, UAS-GFP/ Mtch-IR (BL38986) |
| F,F’ | hs-flp;; His2Av-mRFP, FRT2A*/ MtchΔ* FRT2A |
|  |  |
| **Fig S3** |  |
| B,B’,B’’ | +;+;Mtch2xHA;+ |
| C | hs-flp; Mtch-IR (v106696); Actin (CD2) GAL4, UAS-DsRed/Mtch2xHA |
| E,F,G | +;+;Mtch-2xHA/Vps13D-3xFLAG;+ |
| H | NP1-GAL4/UAS-GFP and NP1-GAL4/UAS-Mtch-IR (v106696) |
|  |  |
| **Fig S4** |  |
| A,A’ | hs-flp; His2Av-mRFP, FRT2A*/ MtchΔ* FRT2A |
| B,B’’ | hs-flp;; His2Av-mRFP, FRT2A*/ Vps13D [MI11101]* FRT2A |
| C,C’ | hs-flp;; His2Av-mRFP, FRT2A*/ MtchΔ,Vps13D [MI11101]* FRT2A |
|  |  |
| **Fig S5** |  |
| A,A’ | hs-flp; Parkin-IR (BL104363); Actin (CD2) GAL4, UAS-DsRed/Mtch2xHA |
|  |  |
| **Fig S6** |  |
| A,A’ | hs-flp; NP1-GAL4/UAS-hBNIP3-HA; Ub-GFP FRT2A/ BNIP3*Δ* FRT2A |
| C,C’ | hs-flp; NP1-GAL4/UAS-hBNIP3-HA; Ub-GFP FRT2A/ Parkin*Δ* FRT2A |
| F,F’ | hs-flp; NP1-GAL4/UAS-hBNIP3-HA; Ub-GFP FRT2A/ *MtchΔ,* Parkin*Δ* FRT2A |
| H,H’,H’’ | hs-flp; Zuc-3xFLAG;His2Av-mRFP, FRT2A*/ MtchΔ* FRT2A |
|  |  |
|  |  |
|  |  |
|  |  |
|  |  |
|  |  |
|  |  |
|  |  |
|  |  |
