# Supplementary material for: The conserved outer mitochondrial membrane protein Mtch regulates mitophagy during Drosophila intestinal development
Source: PLoS Biol. 2026 Jan 23;24(1):e3003616. doi: 10.1371/journal.pbio.3003616 (PMC12829841; doi:10.1371/journal.pbio.3003616)
Supplement: S4 Table — (DOCX) [file pbio.3003616.s010.docx]

| **S4 Table.** Oligonucleotides and gBlocks. | |
| --- | --- |
| gblock synthesized for Mtch deletion | TTAATTTCCACAATTCGACGCACTTACCGAAAAGTCGTGCCGATGTAAGAAATGTTCAACAAATACCTATACATGTTATAGAGCACGACCCACAACCACCATCCATATCGCTAGAGATGGGCTTGGGCAGCTGGTTGATCTACACTCCTAGAGGTGGGATTGTAAATTATGTGTTGAATAATCAAAAAAGCAACATTTAAAATAATTAAAAAAGCTGTATATCAAGAAAGCAACTATTTTTTATGCAACTCCCTGCAAAAACACTTTCTACGGCCAAGCTCTTTTTGGGCCAGAGAATGAGAAGATATCGATTAATCGATGACGAATCGGTTCACGGCATTAGGCAACAACAAAGTACCCGGCCACATTGCTGTCAGAAGTATATTGTTTTGTAATAAAAAAATATAATTTTGCAACTTGCCAAAGCCTTGCAACCCTGTAAGAGACGGCCAACGGTCTAATACTTTTTCTGTCTTTTGCACAGCAGTCCTCCAAATTAAGCTCCGGTATGACAAGCTTCAATCCAAGGCAACTGCATTTTTCTACTGTCTCCAAGCCCAGCCATAATTCTGTTAATACAAGTTTAGATCTACATTGAAGTGGATTTTCATCGTGGTCCGTTAGTCTTGCGCGATGTTTTCTTTTCTTTCAGCAACTACGAGCACTGAAATCGTGAAAAAATCTTTCCCAATCTTGTGCAGACTCCGATATAATTGTTGTTATTGTTTACTGCAAAATTTTGGAAATAAATCGTTTTGCCCTACTAGTGGAACTTTTCCATATATATGTTTTTTTAATTATAATGGTCTACTGGTAACGCTCTTATCGTTACCTTATTTTATTTTTAAGTAACACGTATTAAAGCTCAGGAGTTAAATAAGACATATGGTTTCATTGAAACCCCACTTTTTTAAAGTAGATTTTATATTTACTTTGAATAGTTTTGTTGCGCAATTTATATAGTACTGAAAAAAATATTTTCCTGTTGGAGCCATTAAAA |
| gblock synthesized for 2xHA-Mtch | TACGAACCAATCGCTCCTCTGCCCGGCAAGTCGATACTGGGCAAGCCGATCATGAAGTTGCCGAACATCTTTCAGTACGGTAGGTACAAAGTTCCTTTTTGCCGAAACATAACTAATATTTCCGCTGCGGCTCCTGCAGCTGGCCACATTCGGCGGATCGACGGCTTCTACGGTTGCTACCGCGGTCTAGCTCCTAAATTGGTGGGTTCTCTGGTGGCCATGGTGGTAAGCGACCGAGTGGCCGATCAACTGGGCCTGGAGCAGCCAGAGGAGAATAAGGACGACTCGCAGCTTAGTGATGAGGAATTGCAAGTCTTTGATTTTCAAGTGCCCAAGAATCTCCGTACAACTCCTGATTTGTTTTCTTACAGGTACGTCCAGTTCAAGAAAAGTCTGAAGCGGGATATTGTTTTGATGGTGAGCGGAGTCGTGGCCTCGCATCCTTTTCACGTAATCTCGTTGCGCATGATGGCGCAGTTTGTCGGCCGCGAGACCTTGTACACCTCTATTGTGGGCTCCGTGGCCGAGATTTGGAAGTCTGAGGGAATAGCGGGATTTTTCGCCGGTCTTGTGCCAAAGCTGCTGGGCGATTTGGCCTGCCTAGTGCTCAGCAGCTCCACGATCTACATCCTTAACAAATATATCATCAAGGACAAGTTAGGCAGGCAATACAATTCCGGATTCACACAGTTTGCCGTATCTAGTTTACTGTATCCACTGCAAGTGGTGTCCACCTGCTCAGCGGTAAGTGGCTCACGCCTGATGGCTGCCCAACCGCCAATTATGCCGGCCTACAGGAACTGGGTAGACTGTTGGAATGATTTGCAGGTTCGCGGGGAGCTTAAGCGCGGCAGCTCCCTTTTCTGGCGGTGGGTGTTACAGTGAACTTTATGTTTAAATTAGTAACTACAAAAAACGTCGACTTTCCAGGTCACAATCCATCAGTTCTCCAGTAATAGCCACCTCATTCGCGCCCTTGCCTAAGCTcGCGCGTTACCAGTAGCCGTCAACAACGAGGCTGGACGACCACGGACaGGCTCCGGTATGACAAGCTTCAATCCAAGGCAACTGCATTTTTCTACTGTCTCCAAGCCCAGCCATAATTCTGTTAATACAAGTTTAGATCTACATTGAAGTGGATTTTCATCGTGGTCCGTTAGTCTTGCGCGATGTTTTCTTTTCTTTCAGCAACTACGAGCACTGAAATCGTGAAAAAATCTTTCCCAATCTTGTGCAGACTCCGATATAATTGTTGTTATTGTTTACTGCAAAATTTTGGAAATAAATCGTTTTGCCCTACTAGTGGAACTTTTCCATATACCGATATAATTGTTGTTATTGTTTACTGCAAAATTTTGGAAATAAATCGTTTTGCCCTACTAGTGGAACTTTTCCATATATATGTTTTTTTAATTATAATGGTCTACTGGTAACGCTCTTATCGTTACCTTATTTTATTTTTAAGTAACACGTATTAAAGCTCAGGAGTTAAATAAGACATATGGTTTCATTGAAACCCCACTTTTTTAAAGTAGATTTTATATTTACTTTGAATAGTTTTCAGTTAAGGTTTAATTTCTGCTATATTTTTATGTATATAATACAAATTATCTTATTGATTGTTGATTGATCCTTACACGATTTTTGCAGCCCAATAAAAGCTTAATTAATTAATTTGTCCTGTGCTAACTATAATGCACTCTAGTATCATTTTTTGTAATATTTATACTTTGCTGTTACTACATCTCCAGTACCACCAAGCAAACAAATTTTGCATCTACAACAAATTCTTATTTGTAA |
| gRNA #1 for *Mtch* deletion | AGGCTACAGAGTTGGGCCCTCGG |
| gRNA #2 for *Mtch* deletion/2xHA-Mtch | CGAGGCTGGACGACCACGGACGG |
| Forward Primer for screening *Mtch* deletion | ACTTTCTACGGCCAAGCTCT |
| \| Reverse Primer for screening *Mtch*  deletion \| \| --- \| | TCAGTGCTCGTAGTTGCTGA |
| Forward Primer for screening 2xHA-Mtch | CCAATTATGCCGGCCTACAG |
| Reverse Primer for screening 2xHA-Mtch | AGGTAACGATAAGAGCGTTAC |
| Forward primer to screen *ParkΔ* mutant | TTGCAATTTGGAGGGAAAAC |
| Reverse primer to screen *ParkΔ* mutant | TATTGTCGTGGCATCGCTTA |
| Forward primer to screen *BNIP3Δ* mutant | ACCCCATTTGGCTTTAGCTT |
| Reverse primer to screen *BNIP3Δ* mutant | TGGACTCTTGGGGCTATCAC |
| gRNA #1 for 2xHA-Mtch | ATTCGCGCCCTTGCCTAAGCTGG |
| Forward Primer for Vps13D (qPCR) | CCATGTCGGTTCACGGATTAC |
| Reverse Primer for Vps13D (qPCR) | GTGATCATGTGCGGACTAGTAGC |
| Forward Primer for RPL32 (qPCR) | TGCTAAGCTGTCGCACAAATG |
| Reverse Primer for RPL32 (qPCR) | TCGATCCGTAACCGATGTTGG |
